# Zscan4 Is Regulated by PI3-Kinase and DNA-Damaging Agents and Directly Interacts with the Transcriptional Repressors LSD1 and CtBP2 in Mouse Embryonic Stem Cells

**DOI:** 10.1371/journal.pone.0089821

**Published:** 2014-03-03

**Authors:** Michael P. Storm, Benjamin Kumpfmueller, Heather K. Bone, Michael Buchholz, Yolanda Sanchez Ripoll, Julian B. Chaudhuri, Hitoshi Niwa, David Tosh, Melanie J. Welham

**Affiliations:** 1 Centre for Regenerative Medicine and Departments of Pharmacy & Pharmacology, University of Bath, Bath, United Kingdom; 2 Department of Biology and Biochemistry, University of Bath, Bath, United Kingdom; 3 Department of Chemical Engineering, University of Bath, Bath, United Kingdom; 4 RIKEN Centre for Developmental Biology, Kobe, Hyogo, Japan; University of Minnesota, United States of America

## Abstract

The Zscan4 family of genes, encoding SCAN-domain and zinc finger-containing proteins, has been implicated in the control of early mammalian embryogenesis as well as the regulation of pluripotency and maintenance of genome integrity in mouse embryonic stem cells. However, many features of this enigmatic family of genes are poorly understood. Here we show that undifferentiated mouse embryonic stem cell (ESC) lines simultaneously express multiple members of the Zscan4 gene family, with Zscan4c, Zscan4f and Zscan4-ps2 consistently being the most abundant. Despite this, between only 0.1 and 0.7% of undifferentiated mouse pluripotent stem cells express Zscan4 protein at a given time, consistent with a very restricted pattern of Zscan4 transcripts reported previously. Herein we demonstrate that Zscan4 expression is regulated by the p110α catalytic isoform of phosphoinositide 3-kinases and is induced following exposure to a sub-class of DNA-damage-inducing agents, including Zeocin and Cisplatin. Furthermore, we observe that Zscan4 protein expression peaks during the G2 phase of the cell cycle, suggesting that it may play a critical role at this checkpoint. Studies with GAL4-fusion proteins suggest a role for Zscan4 in transcriptional regulation, further supported by the fact that protein interaction analyses demonstrate that Zscan4 interacts with both LSD1 and CtBP2 in ESC nuclei. This study advances and extends our understanding of Zscan4 expression, regulation and mechanism of action. Based on our data we propose that Zscan4 may regulate gene transcription in mouse ES cells through interaction with LSD1 and CtBP2.

## Introduction

Embryonic stem cells (ESCs) self-renew and are pluripotent, meaning they can differentiate into all cells comprising an adult organism [1]. These properties have made ESCs an attractive source of differentiated cell types for use in both drug discovery and regenerative medicine. While the potential of ESCs has been widely recognized, it is imperative that the mechanisms regulating their self-renewal, pluripotency and stability are better understood, to ensure their efficacy and safety.

The extrinsic factors, signaling pathways and transcription factor networks that contribute to maintenance of mouse ESC self-renewal and pluripotency, referred to as the ‘ESC state’, have been extensively studied [1], [2], [3], [4]. Leukaemia inhibitory factor (LIF) and Bone morphogenetic protein 4 (BMP4) are the key cytokines required for maintenance of ESC self-renewal in culture, acting via the Jak-Stat3 and Smad-Id pathways respectively [5], [6], [7]. Inhibition of glycogen synthase kinase 3 (Gsk-3), which mimics both activation of the Wnt pathway and growth factor-induced PI3K signaling, can enhance mouse ESC self-renewal [8], [9] and assist in maintaining the ‘ground state’ of mouse ESC pluripotency [10], [11], [12]. Inhibition of MAPK signaling, in addition to Gsk-3 inhibition (referred to as 2i conditions) is sufficient to maintain self-renewal of mouse ESCs in the absence of additional exogenous factors [10]. Phosphoinoside 3-kinase (PI3K) signaling has also been implicated in the maintenance of both mouse [13], [14], [15] and human ESC [16] pluripotency.

Oct4, Sox2 and Nanog are amongst the most important transcription factors that contribute to regulation of ESC pluripotency, often referred to as the ‘core transcription factors’ or ‘master regulators’ [1], [17]. Other transcription factors work in concert with these core factors and include Zfx [18], Klf 2 & 4 [19], c-Myc [20], Esrrb [21] and Tbx3 [13], [21]. In addition, epigenetic regulation has been proposed to play an important role in control of the ESC state [1], [22], [23].

Recently, members of the Zscan4 family of zinc finger proteins have been identified as important contributors to the maintenance of the pluripotent state of mouse ESCs [24], [25]. The Zscan4 family comprises 9 very closely related gene paralogues located on mouse chromosome 7 [24], [26]. Full-length Zscan4 proteins comprise an N-terminal SCAN domain of approximately 160 residues and 4 zinc finger motifs, located towards the C-terminus [24], [26]. SCAN domains can mediate dimerisation of other Zinc finger-containing proteins [27], [28], [29], although to date this has not been demonstrated for the Zscan4 family.

In terms of the functional role of Zscan4 family members, Falco et al., first identified Zscan4d as a gene upregulated during zygotic genome activation in mouse 2-cell stage embryos [26]. Blockade of Zscan4d expression during pre-implantation development led to a delay in 2-cell to 4-cell progression and blastocysts that did develop were unable to implant or proliferate in culture [26]. Interestingly, in mouse ESCs Zscan4 mRNA exhibits a very unusual pattern of expression, restricted to only a small proportion of cells [26], [30]. Studies have suggested that Zfp206 and Smad4 play positive and negative roles respectively in regulating Zscan4 expression [31], [32]. We first identified Zscan4c as a gene whose expression is down-regulated in mouse ESCs following inhibition of PI3K signaling [24] and demonstrated that knock-down of Zscan4 family members decreased self-renewal of mESCs, consistent with a role for Zscan4 proteins in maintenance of the ESC state [24]. More recently it has been reported that Zscan4 plays a key role in maintaining the stability and integrity of the ESC genome [25]. Furthermore its transient expression can enhance both the efficiency of generation of induced pluripotent stem cells [33] and genome stability during the reprogramming phase [34]. More recently, Zscan4 has also been shown to be capable of restoring developmental pluripotency to embryonic stem cells [35]. These findings suggest that the Zscan4 family plays multiple roles in maintenance of the ESC state.

To increase our understanding of the biology of the Zscan4 family of proteins we have investigated the patterns and regulation of expression of Zscan4 genes and proteins in mouse ESCs and probed the mechanisms of action of Zscan4. Here we report that multiple Zscan4 genes are expressed in different ESC lines, including genes previously proposed as pseudogenes. In multiple pluripotent mouse stem cell lines Zscan4 protein expression was consistently found to be highly restricted, with between 0.1 and 0.7% Zscan4-positive cells detected. We also show that Zscan4 protein levels are modulated by PI3K and Gsk-3-dependent signaling, in response to DNA damage and during the G2 phase of the cell cycle. Consistent with a role as a potential regulator of transcription, we demonstrate that Zscan4c modulates transcription in a heterologous system and that Zscan4 proteins are located in the nucleus, where they interact with components of co-repressor complexes, including LSD1 and CtBP2. Based on our current understanding of Zscan4 biology we believe that this gene family represents an intriguing new paradigm for the control of the ESC state.

## Materials and Methods

### Culture of mouse pluripotent cells

The mouse ES cell lines E14tg2a [36], E14 Gsk-3 double knock-out (DKO; [37]), CCE, CGR8, IOUD2, R1 and ZE3-MC-1 [25] and the mouse iPS cell line [38] were cultured as described previously [8], [14], [39].

### Generation of GFP-Zscan4c and Zscan4c-V5-His mouse ES cell lines

An eGFP-Zscan4c fusion protein was generated by amplifying the Zscan4c coding sequence with the primers: forward (5′-TTATGGCTTCACAGCAGGCA) and reverse (5′- TTAATTGCGGCCGCTCAGTCAGATCTGTGGTAAT). The resulting product was cloned into SmaI/NotI digested pTRE-Tight (Clontech) which contained the coding sequence for eGFP. This generated an in-frame N-terminal fusion between eGFP and Zscan4c. To generate ESCs with eGFP-Zscan4c under the control of Tet-on regulation, R1 mESCs containing the Tet-On Advanced Transactivator (Clontech; a kind gift of Giusi Manfredi, University of Bath) were co-transfected by electroporation with pTRE-eGFP-Zscan4c and a vector conferring resistance to Hygromycin B. Clones were selected in 300 µg/ml Hygromycin B, expanded, screened for expression and further characterised (Supplemental Figure S1). The karyotype of clones used for further analyses was checked and confirmed as normal (data not shown). To generate ESCs expressing an inducible c-terminal V5-His epitope tagged version of Zscan4c under the regulation of the Tet-off system, Zscan4c-V5-His was PCR amplified from a pcDNA3.1vector containing the required insert [24] using the primers forward (5′-AATGTCGACTAATACGACTCACTATAGGG) and reverse (5′-AATGTCGACTAGAAGGCACAGTCGAGG). This product was blunt-end ligated into the Tet-off response plasmid pUHD10-3 [40] to create the vector pTet-off-Zscan4c-His-V5. To generate inducible Zscan4c-Tet-off ESC lines, linearized pTet-off-Zscan4c-His-V5 was electroporated as previously described [14] into E14tg2A (clone R63) ESCs which constitutively express the tetracycline transactivator (tTA) driven by the CAG promoter [41]. Transfectants were selected in G418 and surviving clones expanded and screened by immunoblotting for the induction of Zscan4c expression after withdrawal of tetracycline. Clones with the highest degree of inducible expression were used in this study and further characterised (Supplemental Figure S2). Clones were routinely cultured in the presence of 1 µg/ml tetracycline to maintain transgene expression switched off, tetracycline was removed to induce Zscan4c-V5-His expression.

### Generation of mouse ES cell lines expressing myristyolated p110α

A version of p110α containing an in-fame N-terminal N-myristoyl transferase recognition peptide sequence, MGSSKSKPK, was amplified by PCR from the plasmid pPyCAG-myrp110α-IP [42] and subcloned into the piggyBac vector pPBCAGcHAIN to generate pPB-myr-p110α. One day prior to transfection, 2×10^4^ OCRG9 mouse ESCs (Rex1-GFP/Oct3/4-CFP double knock-in ES cells)/well were plated into 12-well cell culture trays. The following day, 1 µg of pPB-myr-p110α and 1 µg transposase expressing helper plasmid (pCAG-PBase) were mixed in 25 µl of GMEM without serum. 25 µl of diluted Lipofectamine2000 (2 µl Lipofectamine2000 plus 23 µl GMEM) was added to the DNA mix and incubated at room temperature for 10 min. 450 µl of GMEM plus serum was then added and the mixture applied to prepared cells. After 3 hours, the medium was replaced with fresh medium. Selection with G418 was initiated after a further 24 h and colonies generated after 6 days in selection were picked and expanded.

### RNA isolation and RT-PCR

RNA was isolated and RT-PCR performed as previously described [43]. The list of primers used in this study are summarised in Table S1.

### Cloning and sequencing of Zscan4 transcripts

RNA was isolated and RT-PCR performed as above using the following primers (Forward 5′-ACAATGGCTTCACAGCAGG, Reverse 5′-ACGATGGTAAGTGGATGATTGG) and limited amplification (22 cycles). PCR products were TOPO cloned into pcDNA3.1 (Invitrogen). Clones were picked, plasmid DNA extracted and 96 insert verified clones were sequenced using the primer 5′-CATCCTAGAACATTCTTCACAC. All primers used bind to all 9 Zscan4 paralogs with equal efficiency. Sequences were analysed using Sequencher (GeneCodes).

### Generation of transcriptional reporters

A synthetic GAL4-UAS promoter, containing five GAL4 bindings sites (5′-CGGAGTACTGTCCTCCG-3′), was cloned into the pGL4.26 luciferase reporter (Promega), using the synthetic sequences: forward 5′- CCGGAGTACTGTCCTCCGTACGGAGTACTGTCCTCCGTATGCCGGAGTACTGTCCTCCGATCGGAGTACTGTCCTCCGTATGCCGGAGTACTGTCCTCCGC & reverse 5′-TCGAGCGGAGGACAGTACTCCGGCATACGGAGGACAGTACTCCGATCGGAGGACAGTACTCCGGCATACGGAGGACAGTACTCCGTACGGAGGACAGTACTCCGGGTAC, as shown in Supplemental Figure S3. The trans-activator reporter plasmid, pFA-CMV (Stratagene), was used to create a series of Zscan4c-Gal4 fusion proteins using the primer sequences listed in Supplemental Table S2. A Nanog Gal4-fusion was also generated. After sequence verification of the constructs, transcriptional activity was tested in heterologous Hek293 cells. For transfection, cells were plated at 60,000 cells/well in a 96-well dish and incubated for 24 h in 100 µl DMEM plus 10% (v/v) FBS, which was subsequently replaced with 50 µl of the same medium. Following this, 100 ng pGL4.26 GAL4-UAS, 20 ng pFA-CMV SCAN (or SCAN+ or SCAN+ZnF) and 20 ng phRL-TK (Renilla plasmid to control for transfection efficiency) were combined in 12.5 µl Optimem and incubated at room temperature for 5 min. The DNA was then combined with diluted Lipofectamine 2000 (0.5 µl in 12.5 µl Opti-Mem) and incubated for 20 min before addition to cells. After 24 h luciferase detection was carried out using Dual-Glo Luciferase Assay System (Promega – E2920). After subtraction of background fluorescence for Renilla and Firefly luciferase, the ratio of reporter (firefly) luminescence to control (Renilla) luminescence was calculated and values were normalized relative to Gal DBD response alone – this value represents the Relative Response Ratio.

### Generation of mouse pan-Zscan4 antibodies

Polyclonal rabbit antisera were generated against a Zscan4-specific peptide (GVPQDSTRASQGTSTC, amino acids 322–337; anti-pan Zscan4) by Millipore/Merck according to company procedures. Specificity for Zscan4 was confirmed by immunoblotting in the presence and absence of blocking peptide and immunoprecipitation (Figure S4).

### Immunofluorescence and flow cytometry

Cells were fixed with 4% (w/v) paraformaldehyde for 20 min, permeablised in 1% Triton X-100 for 20 min, blocked for 1 h in 1% blocking reagent (Roche), washed and incubated with 1∶500 dilution of anti-Zscan4 antisera. After washing, a 1∶100 dilution of anti-rabbit-Texas Red antibody (Vector Laboratories) was added. After a second round of washing DAPI was added for 20 min. Samples were again washed and mounted under a coverslip with fluorescence mounting medium (DAKO). Fluorescence was visualised using Leica DMI 4000B fluorescence microscope or Zeiss LSM Meta Confocal Microscope (Bioimaging Suite, University of Bath, UK). For cell cycle analyses by flow cytometry, cells were trypsinised and fixed with 70% (v/v) ice-cold ethanol. After washing with PBS and re-hydration for 10 min, cells were incubated with 7-AAD (0.5 mg/ml) for 1.5 h at 4°C. A minimum of 10,000 cell events were acquired using FACS Canto flow cytometer (Becton-Dickinson) and analysed using FACS Diva software. Alternatively, live cells were stained with Nuclear-ID Red according to the manufacturer's instructions (Enzo Life Sciences) and a minimum of 140,000 cell events were analysed as above.

### Protein extraction, immunoprecipitation and immunoblotting

Cytosolic or nuclear cell extracts were prepared as described previously [44] and protein concentrations determined. To generate total cell extracts RIPA lysis buffer (150 mM NaCl, 50 mM TrisHCl pH8, 1% (v/v) NP40, 0.5%(w/v) Na Deoxycolate, 0.1% (w/v) SDS, 25 U/ml Benzonase, 1 mM sodium vanadate, 1 mM sodium molybdate, 10 mM sodium fluoride, 40 µg/ml PMSF, 0.7 µg/ml Pepstatin , 10 µg/ml Aprotinin, 10 µg/ml Leupeptin, 10 µg/ml Soyabean trypsin inhibitor) was used. For immunoprecipitation, equal amounts of protein (cytosolic or nuclear extracts) were pre-cleared with protein A sepharose beads and then precipitated with either Nanotrap beads (30 ml of 50% (v/v) slurry, Chromatek), pre-immune serum (5 ml), anti-panZscan4 antiserum (5 ml), anti-LSD-1 (2 µg per sample, Abcam, ab37165), anti-CtBP2 (2 µg per sample, BD Transduction Labs, Cat No. 612044) or anti-V5 epitope (2 µg per sample, Abcam ab27671) antibodies and immune complexes captured on protein A or G sepharose beads, prior to extensive washing with nuclear extraction buffer and boiling in Laemmli buffer. For immunoblotting, 20 µg of each protein sample or the entire immunoprecipitate was separated by SDS-PAGE and transferred to nitrocellulose as previously described [45]. Immunoblotting was carried out with the following primary rabbit polyclonal antibodies: 1∶4000 anti-panZscan4 (Merck Millipore, AB4340); 1∶2000 LSD-1 (Abcam, ab37165); 1∶5000 GFP (MBL, 598), or mouse monoclonal antibodies at 1∶2000 anti-CtBP2 (BD Transduction Laboratories, 612044), 1∶5000 anti-V5 epitope (Abcam, ab27671), 1∶1000 γ-H2AX (Merck Millipore clone JBW301) or 1∶20000 anti-GAPDH (Ambion, AM4300). Anti-mouse or anti-rabbit secondary antibodies conjugated to horseradish peroxidase (DAKO) were used for detection and blots were developed using ECL Prime according to the manufacturer's instructions (GE Healthcare). Protein relative quantification was carried out using ImageQuant RT-ECL imager and analysed using ImageQuant TL software (GE Healthcare). Blots were stripped and reprobed as previously described [45].

### Affinity purification of Zscan4 interacting proteins

GFP-Trap-A beads (Chromotek) provide a means of high affinity purification in a single step, facilitating rapid precipitation and the potential to capture less stable interactions. Following induction of eGFP-Zscan4 expression by addition of Dox to GFP-Zscan4c ESCs for 24 h, cells were harvested, washed in PBS and cell pellets resuspended in cytosolic extraction buffer (20 mM HEPES, 10 nM KCl, 1 mM EDTA, 10% (v/v) Glycerol, 1 mM PMSF, 1 µg/ml Aprotinin, 1 µg/ml leupeptin, 1 µg/ml pepstatin, 5 µg/ml antipain, 157 µg/ml benzamidine, 5 mM β-glycerophosphate, 5 mM sodium fluoride, 1 mM sodium orthovanadate, pH 7.9). After a 30 min incubation at 4°C, the suspension was drawn through a 27G needle ten times and centrifuged at 163000× g for 5 min at 4°C. The supernatant was removed and designated ‘cytosol’. The remaining nuclear pellets were washed twice with ice cold cytosolic extraction buffer before being resuspended in nuclear extraction buffer (20 mM HEPES, 10 nM KCl, 400 mM NaCl, 1 mM EDTA, 20% (v/v) Glycerol, 1 mM PMSF, 1 µg/ml Aprotinin, 1 µg/ml leupeptin, 1 µg/ml pepstatin, 5 µg/ml antipain, 157 µg/ml benzamidine, 5 mM β-glycerophosphate, 5 mM sodium fluoride, 1 mM sodium orthovanadate, pH 7.9). Following rotation at 4°C for 1 h samples were centrifuged at 163000× g for 15 min. The supernatant was collected and designated as the nuclear extract. Cytosolic and nuclear fractions were cleared by centrifugation at 60,000× g for 30 minutes at 4°C. A subsequent buffer exchange to immunoprecipitation (IP) buffer (50 mM Na_2_HPO_4_, 150 mM NaCl, pH 7.4) was performed using Amicon centrifugal filters with a 10 kDa cut-off according to the manufacturer's protocol. Prior to immunoprecipitation, lysates were pre-cleared with hydrated sepharose beads for 1 h at 4°C on a rolling shaker. IPs were performed with 500 µl GFP-Trap-A beads (Chromotek) for 1–3 hours on a rolling shaker at 4°C, followed by three washes with IP buffer. Bound protein was eluted with 200 mM glycine at pH 2.5 and 1 M Tris-base (pH 10.4) was added for neutralization. The eluates were centrifuged at 1000 rpm for 1 minute and the supernatant transferred to Amicon centrifugal filters with a 3 kDa cut-off for concentration, according to the manufacturer's recommendations. SDS loading buffer was added and samples were boiled for 5 minutes. Proteins purified from uninduced and induced-derived cell extracts were separated by large format SDS-PAGE, stained with Coomassie blue and proteins enriched in the samples prepared from induced cells were excised from the gel and submitted for protein sequencing at The University of Bristol Proteomics Facility (http://www.bristol.ac.uk/biochemistry/proteomics/services.html). Automated in-gel tryptic digestion was performed using a ProGest unit and resulting peptides analysed by reverse-phase LC MSMS using an LTA-Orbitrap Velos mass spectrometer. The mass spectral data from each fraction was combined prior to database searching using Mascot to identify the proteins present in the sample. Protein scores greater than 64 were classed as significant (p<0.05). In addition, MSMS analyses generated sequence data for a number of peptides. Following these analyses and comparison to the mouse protein sequence database, sequences were returned for 3 out of 4 proteins.

## Results

Members of the Zscan4 family of genes have been implicated in the regulation of pluripotency and genome stability, both functions that impact on maintenance of the stem cell state of mouse ESCs [24], [25]. Two enigmatic features of the Zscan4 family are the existence of 9 highly related gene paralogues and a very restricted pattern of expression. These features suggest Zscan4 family members may regulate the ESC state by novel mechanisms. Our aim was to investigate the underlying basis of the restricted expression pattern observed and further examine the mechanism of action of Zscan4 in ESCs.

### Multiple Zscan4 gene transcripts are expressed in self-renewing mouse ESCs

**Figure 1:**
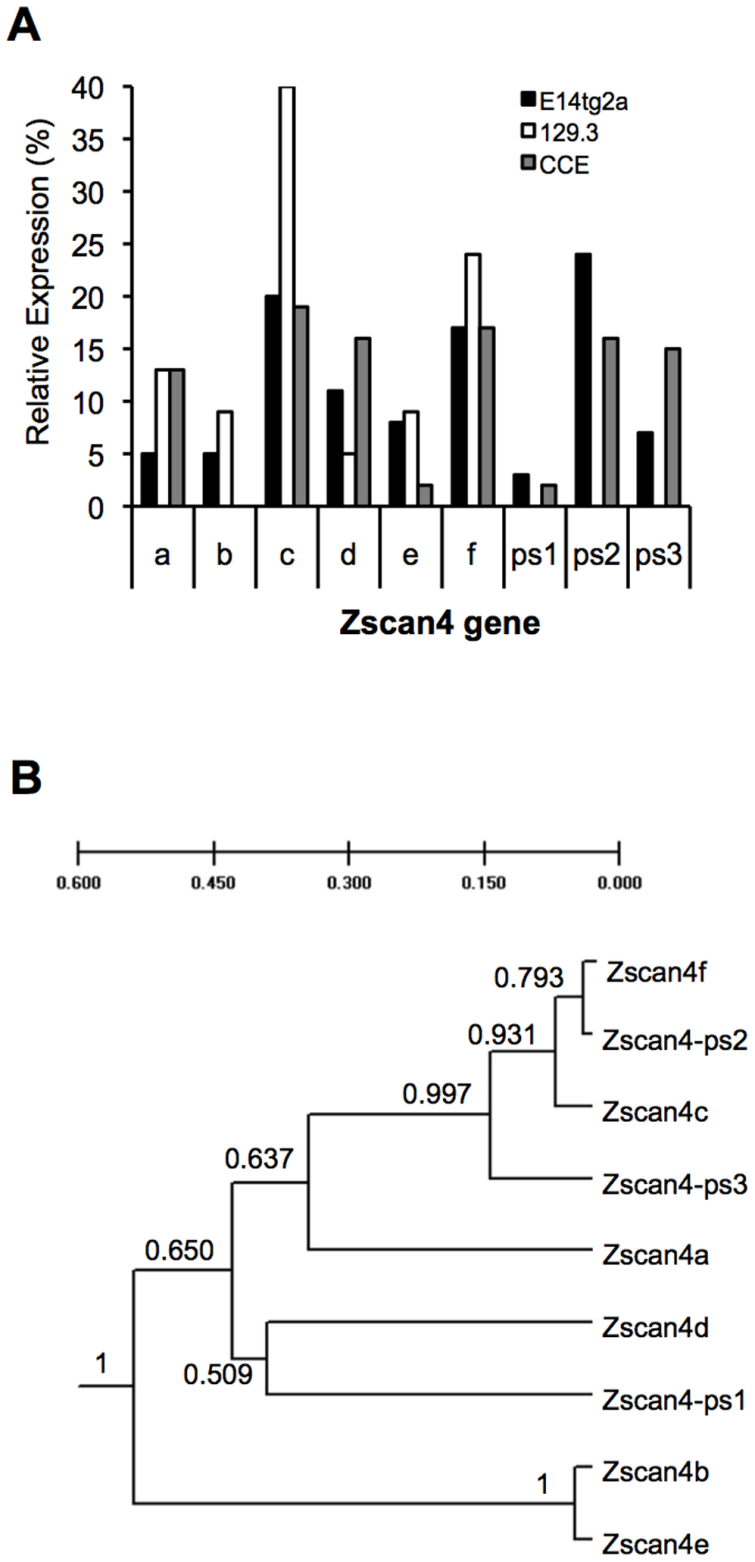
Expression patterns of Zscan4 gene paralogues in mouse ESC lines. **A.** The mouse ESC lines E14 or CCE were cultured in the presence of KO serum replacement and LIF, RNA was extracted, limited amplification by RT-PCR was performed and fragments cloned. 96 independent clones were sequenced and categorised as representing one of the 9 mouse Zscan4 paralogues present in the NCBIm37 database based on SNPs. The relative expression of each Zscan4 paralogue, as a percentage of the total number of Zscan4 gene sequences obtained for E14 and CCE ESCs, are shown in comparison with the 129.3 ESC line, data derived from that presented in [26]. **B.** The suggested evolution of Zscan4 genes in the mouse based on sequence variation. Dendrogram (Unweighted Pair-Groups Method using Averages) showing genetic relationship between Zscan4 paralogs based on Nei's [1972] original distance with 1000 bootstrap replications (confidence values given at each node). The dendrogram was produced using Tools for population genetic analysis (www.marksgeneticsoftware.net).

Individual Zscan4 genes have been reported to be differentially expressed in 2-cell stage mouse embryos and mouse ESCs, with three members, Zscan4-ps1, Zscan4-ps2 and Zscan4-ps3, proposed as pseudogenes [26]. In contrast, we have previously detected Zscan4-ps2 transcripts in undifferentiated E14 ESCs [24]. Given this discrepancy, and the importance of knowing which Zscan4 genes are most highly expressed in ESC, thereby ensuring that the most relevant are studied further, we investigated the patterns of expression of individual Zscan4 genes in two distinct mouse ESC lines. The very high sequence homology of Zscan4 paralogues precluded a PCR-based approach to quantify expression of individual genes and instead ESC cDNA was subjected to a limited number of amplification cycles, using primers that hybridise equally to transcripts from all 9 Zscan4 genes, prior to direct cloning into TOPO vectors. 96 clones were selected at random for DNA sequencing and each individual sequence returned was assigned to one of the 9 Zscan4 genes based on information in the most recent build of the mouse genome on NCBI (NCBIm37), as described in Supplemental Figure S5. Figure 1A shows the relative expression of each Zscan4 transcript in E14 and CCE ESCs, compared to the 129.3 ESC line reported previously [26]. Zscan4c, 4f and ps2 were the most abundant transcripts detected in E14 and CCE ESCs. The most striking difference observed was in the expression of Zscan4-ps2 in both E14 and CCE, compared to its absence in 129.3. Interestingly, phylogenetic analyses of the 9 Zscan4 paralogues (Figure 1B), suggests that Zscan4c, 4f and ps2 are the most closely related family members. In view of this, it is interesting that these 3 genes should consistently be the most abundant transcripts detected, suggesting they constitute the most functionally relevant Zscan4 genes in ESCs. We also detected expression of Zscan4-ps1 and Zscan4-ps3, suggesting their designation as pseudogenes, based on lack of expression, is no longer correct in the light of this new analysis.

### Restricted patterns of Zscan4 expression are a feature of multiple pluripotent mouse stem cell lines

**Figure 2:**
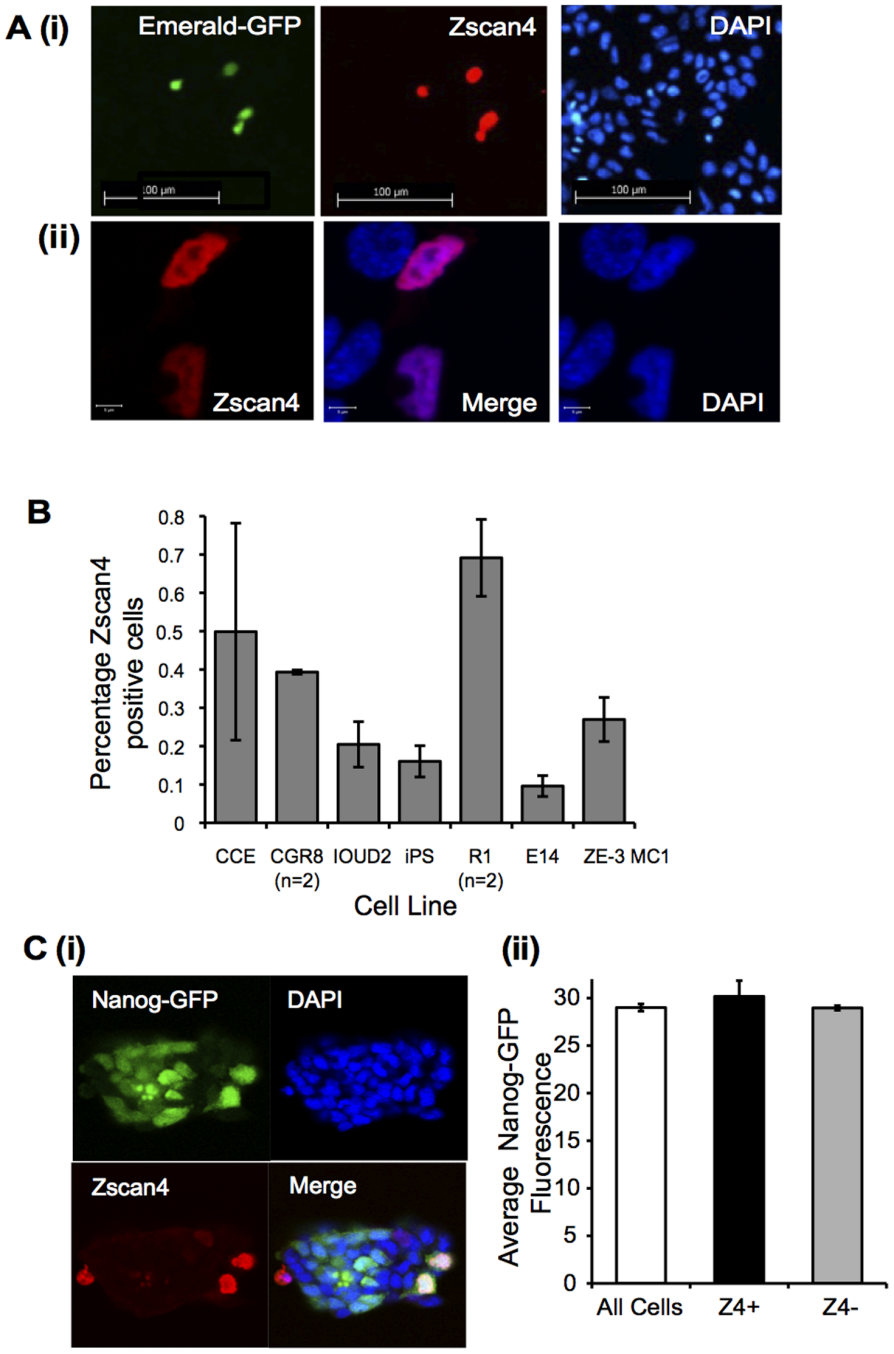
Zscan4 protein expression is highly restricted in mouse ESC and iPSC lines. **A.** The ZE3-MC1 Zscan4 reporter line [25] was cultured in serum replacement plus LIF, cells were fixed and immunostaining was carried out with pan-Zscan4 antibodies (Red). Expression of the Emerald reporter is shown (Green) and cells were counterstained with DAPI (Blue). (**i**) Scale bars = 100 µm. (**ii**) Scale bars = 5 µm. **B.** The mouse ESC and iPSC lines indicated were cultured in the presence of serum replacement plus LIF and subsequently fixed and immunostained with the pan-Zscan4 antibody, while nuclei were counter-stained with DAPI. The proportion of Zscan4 positive cells present were determined by counting several thousand cells for each cell line sample. Data represent average of three independent replicates ± SEM, unless otherwise indicated. **C.** A colony of Nanog-GFP reporter cells stained with the anti-Zscan4 antibody. (**i**) The GFP reporter (Green), Zscan4 (Red) and nuclei (DAPI, Blue) are shown, along with a merged image. (**ii**) Nanog-GFP cells were stained for Zscan4 protein. The levels of Nanog-GFP and Zscan4 protein expression were calculated using Cell-P (Olympus) using fluorescent images captured using an Olympus IX51 epifluorescence microscope. Mean fluorescence intensity for GFP (Nanog) was compared between all cells, Zscan4 expressing (+ve) and non-expressing (−ve) cells.

To date, whole mount *in situ* hybridization of ESC colonies [26] and Zscan4c-based promoter-reporter systems [25] have suggested that expression of Zscan4 transcripts is restricted to between 3 and 5% of cells in a population of undifferentiated mouse ESCs at any one time. We wanted to investigate this highly unusual pattern of expression further and, in particular, determine whether Zscan4 protein shows a similarly restricted pattern of expression to Zscan4 RNAs. To achieve this aim we generated a pan-Zscan4 anti-peptide polyclonal antibody, which detects Zscan4 in immunoblots and is specifically blocked by the immunizing peptide (Supplemental Figure S4A). We initially used ZE3-MC1 ESCs, that express Emerald-GFP under the control of a 2.6 kb fragment of the Zscan4c promoter [25], to measure the correlation between Zscan4 transcriptional reporter and protein expression. As shown in Figure 2A (i) a robust correlation between detection of Emerald-GFP and staining with the Zscan4 antibody was observed, with Zscan4 protein being localized primarily in the nucleus (Figure 2A(ii)). Having established concordance between the Zscan4 reporter and protein expression, we analysed the proportion of Zscan4 positive cells present in populations of undifferentiated mouse ESC lines and a mouse iPSC line. Our data, shown in Figure 2B, demonstrate that the proportion of cells staining positive for Zscan4 protein is between 0.1 and 0.7% (average of 0.33%) lower than the estimated number of cells expressing Zscan4 transcripts reported previously [25], [26]. Even with the ZE3-MC1 reporter cell line generated and used by Zalzman and colleagues [25], we consistently detected 10-fold fewer Zscan4 positive cells than reported previously and we obtained similar results when the expression was measured using flow cytometry (average of 0.43% positive cells, S.E.M 0.03, n = 3, events = 140,000–478,000). Despite these differences, our studies clearly demonstrate that Zscan4 protein expression, like Zscan4 transcripts, exhibits a highly restricted pattern of expression in a range of undifferentiated mouse pluripotent cells, including iPSCs. In further analyses we examined whether there was any correlation between Zscan4 protein expression and the master regulator, Nanog, known to exhibit fluctuations in expression [46]. Using an ESC line expressing GFP under the control of the endogenous Nanog promoter (Nanog-GFP; [46]) no clear correlation between Zscan4 expression and levels of Nanog was observed (Figure 2C).

### Zscan4 gene expression is regulated by the p110α isoform of phosphoinositide-3 kinases

**Figure 3:**
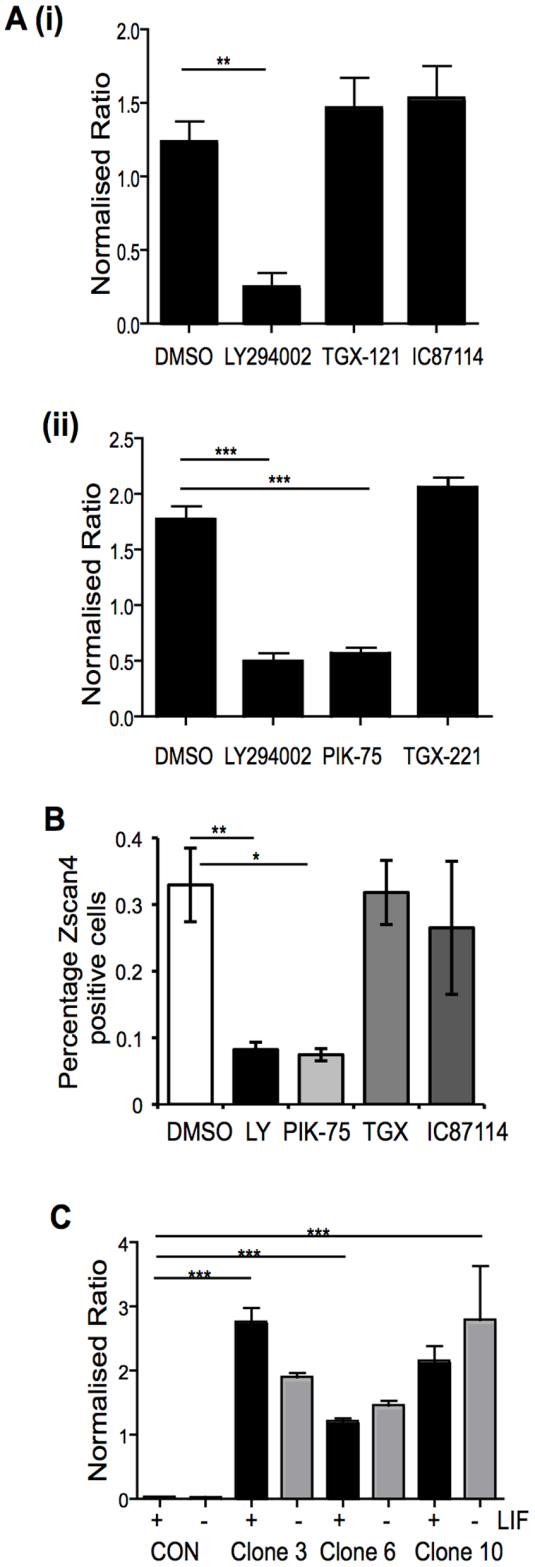
Zscan4 gene expression is regulated by the p110α isoform of PI3Ks. **A.** (**i**) and (**ii**) E14 ESCs were cultured in the presence of LIF, with the addition of DMSO (control) or the inhibitors indicated: 5 µM LY294002 (broad spectrum PI3K inhibitor), 10 µM TGX121 (p110β), 5 µM IC87114 (p110δ), 25 nM PIK75 (p110α), 100 nM TGX221 (p110β), for 48 h. RNA was extracted and levels of Zscan4 expression analysed by qRT-PCR and normalised to levels of β-actin. Mean values are shown with standard deviations (n = 4). **, p<0.005, ***, p<0.0005 in a Student's t-test. **B.** ESCs were cultured in LIF and treated with either DMSO as a control or with 5 µM LY294002 (LY), 10 nM PIK75, 50 nM TGX221 (TGX), or 5 µM IC87114 for 48 h prior to immunostaining for Zscan4. The mean percentage of Zscan4 positive cells with SEM are shown. *, p<0.05, **, p<0.005 following an ANOVA and Tukey's post-hoc test. **C.** ESC clones over-expressing myristoylated p110α catalytic subunit of PI3Ks were cultured in the presence or absence of LIF. As a control parental OCRG9 ESCs were grown in presence and absence of LIF for 4 days. Expression of Zscan4 was analysed by qRT-PCR and Zscan4 expression normalised to levels of GAPDH. The averages and SEM of triplicate samples from each of three independent biological replicates are shown: ***, p<0.0005, in a Student's t-test.

We originally identified Zscan4c as a gene that was rapidly down-regulated upon inhibition of PI3K signaling in mouse ESCs [24]. In this earlier study we had used the broad selectively PI3K inhibitor, LY294002, which targets most PI3K isoforms. Owing to the significant interest in the PI3K signaling pathway as a drug target, small molecule inhibitors, which show selectivity for specific PI3K isoforms, have become available [47]. Mouse ESCs express three of the class 1 PI3K catalytic isoforms, p110α, p110β and p110δ [48]. Therefore, we investigated the coupling of specific p110 PI3K catalytic subunit isoforms to regulation of Zscan4 expression, initially using qRT-PCR. As shown in Figure 3A, inhibition with LY294002 led to a reduction of approximately 80% in Zscan4 transcripts, consistent with previous results [24]. Inhibition of p110β isoform with either TGX121 (Fig. 3A(i)) or TGX221 (Fig. 3A(ii)) or inhibition of the p110δ isoform with IC87114 (Fig. 3A(i)) did not alter Zscan4 RNA levels. In contrast, inhibition of p110α with PIK75 (Fig. 3A (ii)), which is among the most selective p110α inhibitors available to date [49], led to a reduction in Zscan4 expression to the same extent to that observed with LY294002. An un-related p110α inhibitor, compound 15e, also led to a reduction in Zscan4 expression, while the mTOR inhibitor, Rapamycin, had no effect (Supplemental Figure S6). Treatment with LY294002 or PIK-75 also resulted in a significant (3–4-fold) reduction in the number of Zscan4 positive cells present within the treated population (Fig. 3B) and these data were further confirmed by immunoblot analyses (data not shown). To further investigate the regulation of Zscan4 expression by the p110α PI3K isoform, we over-expressed a myristyolated version of p110α (myr-p110α) under the control of the CAG promoter [50] in OCRG9 mESCs. Zscan4 expression was analysed by qRT-PCR in three independent clones over-expressing myr-p110α. Consistent with our loss of function data, expression of p110α led to a significant up-regulation in Zscan4 expression (Figure 3D) and this was observed in cells grown with or without LIF for 4 days.

### Involvement of Gsk-3-dependent signaling in regulation of Zscan4 expression

**Figure 4:**
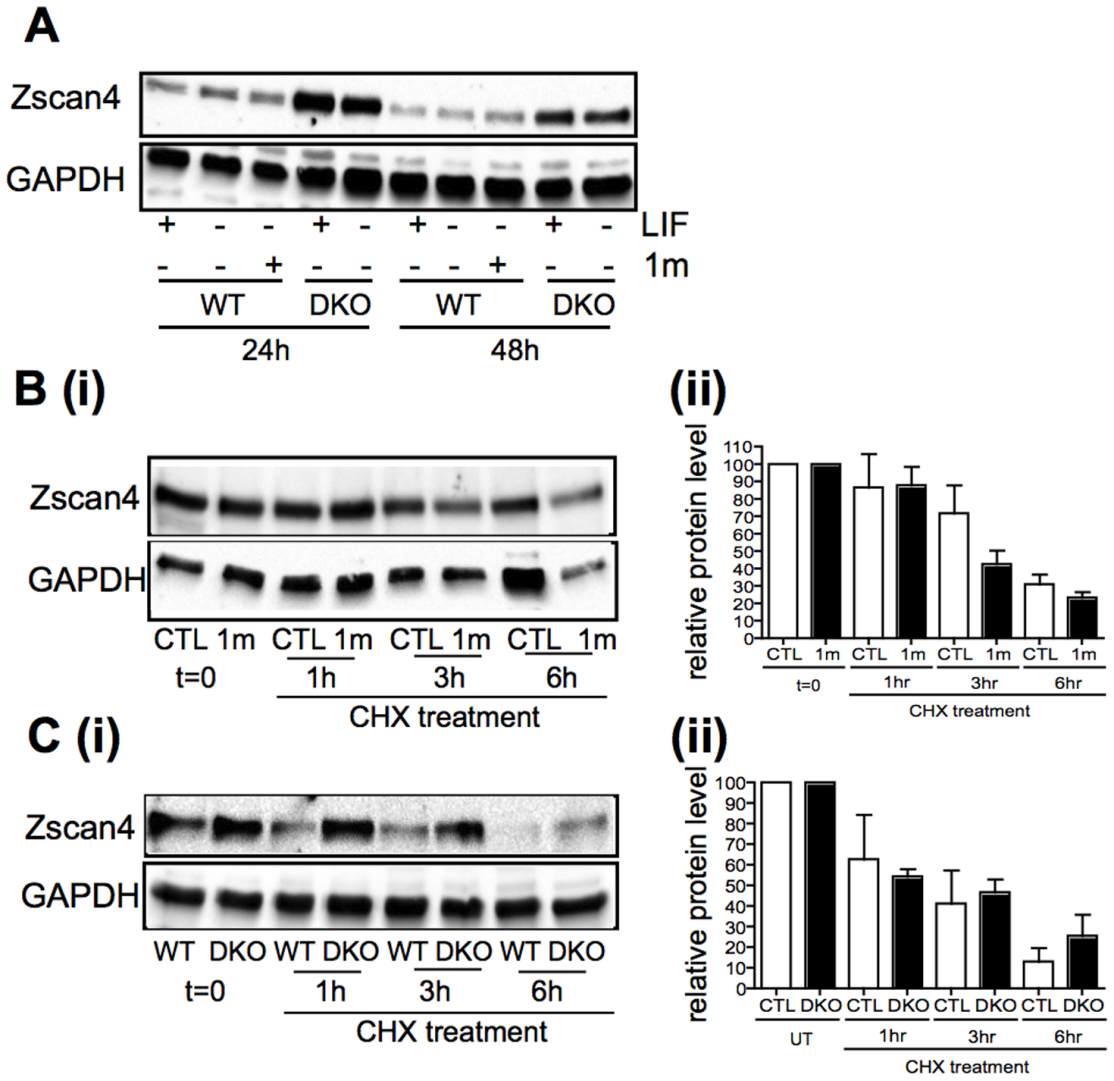
Long-term inhibition of Gsk-3 signalling leads to up-regulation of Zscan4 protein expression. **A.** Wild-type (WT) and Gsk-3 double knock-out (DKO) ESCs cells were grown in the presence or absence of LIF for the times indicated. WT ESCs cultured in the absence of LIF were also incubated with 2 µM 1 m. Protein and RNA were extracted at the times indicated and immunoblotting performed with the indicated antibodies. **B.** WT mESCs (CTL) cultured for 24 h in N2B27 plus LIF and BMP4 with or without 2 µM 1 m, or **C.** WT or DKO ESCs cultured in N2B27 plus LIF and BMP4, were incubated with Cycloheximide (CHX) to halt protein synthesis. (i) Protein samples were extracted after 0, 1, 3 and 6 hours CHX treatment and immunoblotting performed with the indicated anti-Zscan4 or GAPDH antibodies. (ii) Zscan4 protein levels were normalised to GAPDH and a value of 100 was given to the t = 0 samples, to allow direct comparison of half-life values between treatments. The graphs show the average and S.E.M of triplicate experiments.

We next investigated whether other pathways implicated in the coordination of ESC self-renewal and pluripotency also control Zscan4 expression. Inhibition of Gsk-3 has been implicated in the maintenance of ESC self-renewal [8], [9], as well as the ground state of ESC pluripotency [10] and our previous work has demonstrated that short-term treatment with the Gsk-3 inhibitor BIO leads to a small, but significant increase in Zscan4 RNA expression [24]. Here we investigated Zscan4 expression in ESCs in which all 4 Gsk-3 alleles have been knocked out (DKO; [37]) compared to their wild-type parental cells incubated with or without the more selective Gsk-3 inhibitor, 1 m [8]. Interestingly, levels of Zscan4 protein were much higher in Gsk-3 double knock-out ESCs, both in the presence and absence of LIF (Fig. 4A and C), when compared to WT ESCs, while treatment of WT ESCs with 1 m for 24 or 48 h had little influence on Zscan4 expression (see Fig. 4A and B). These data suggest long-term inactivation of Gsk-3 leads to up-regulation of Zscan4 expression and consistent with this we find that culture of WT ESCs in 1 m for 14 days leads to a corresponding increase in Zscan4 RNA expression (data not shown). Having established that long-term inhibition of Gsk-3 results in enhanced levels of Zscan4 protein we wondered whether Gsk-3 plays a role in regulating the stability of Zscan4 protein, as it does for other regulators of pluripotency, including ß-catenin and c-Myc. To assess protein half-life, WT ESCs incubated with or without 1 m (Gsk-3 inhibitor) (Figure 4B) or DKO (Figure 4C) ESCs were treated with cycloheximide for 1 to 6 h. These data suggest that Zscan4 protein has a half-life of between 3–6 h, which is not notably prolonged upon disruption of Gsk-3 activity.

### Zscan4 expression is induced in response to DNA damage

**Figure 5:**
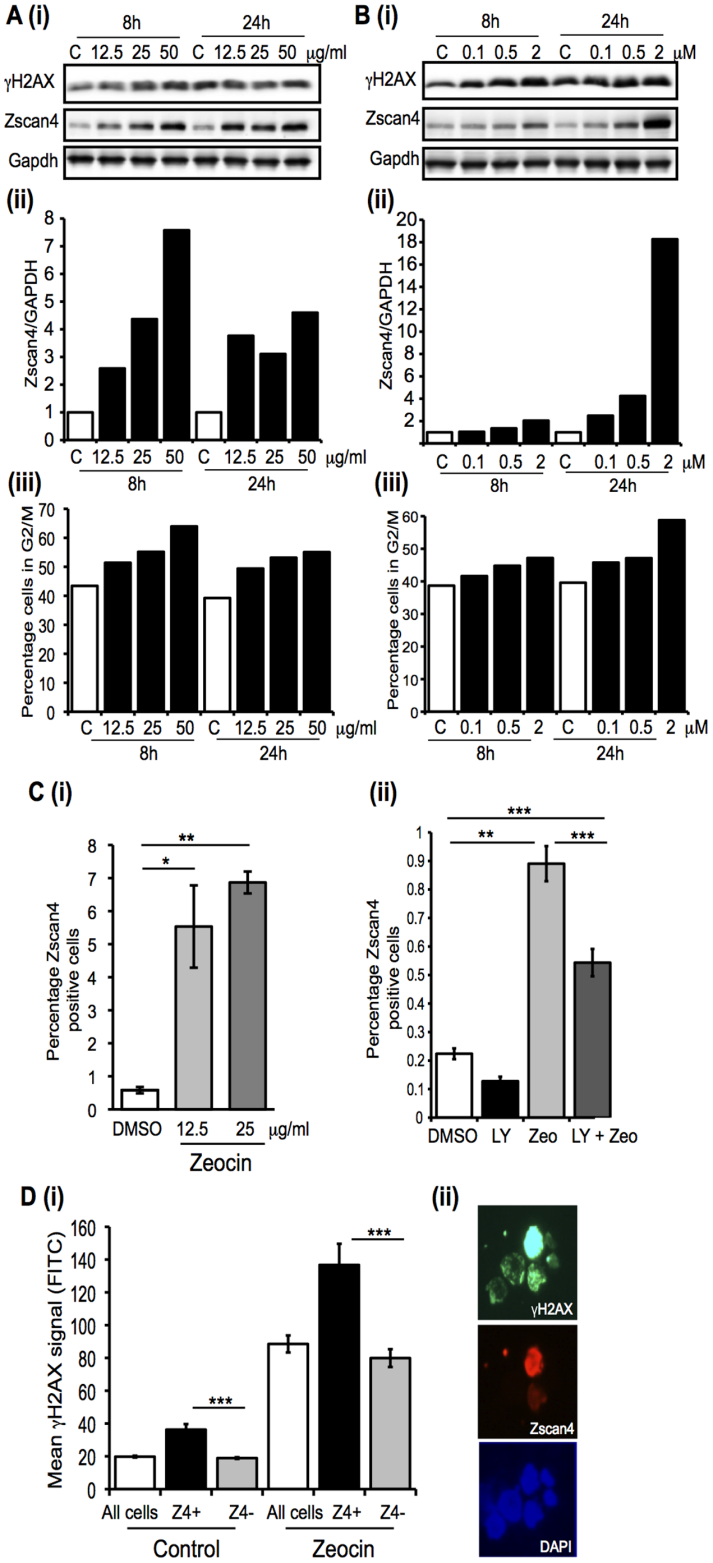
Zscan4 expression is enhanced in response to DNA damage induction. E14 ESCs were plated in the presence of LIF for 4**A.** Zeocin or **B.** Cisplatin for 12 h. After this cells were washed extensively and fresh media added. Following a further 8 or 24 h samples were taken and cells fixed for cell cycle analysis or protein extracted into RIPA buffer. (i) Levels of H2AX phosphorylation at S139 (γH2AX) and Zscan4 protein expression were examined by immunoblotting. (ii) Zscan4 protein expression normalized to GAPDH is shown. (iii) The percentage of cells in G2/M phase of the cell cycle are shown for different samples. **C.** (**i**) The percentage of Zscan4 positive cells were determined after 24 h treatment with the indicated doses of Zeocin. (**ii**) The effect of inhibition of PI3Ks by LY294002 (LY, 5 µM) on the ability of Zeocin to induce Zscan4 expression was examined following 24 h treatment (12.5 µg/ml). In each case the mean and SEM are shown. *, p<0.05, **, p<0.005, ***, p<0.0005 following an ANOVA and Tukey's post-hoc test. **D.** (**i**) E14 ESCs were co-stained for γH2AX and Zscan4 and the mean fluorescence intensity of γH2AX staining determined for Zscan4 positive and negative populations using Cell-P (Olympus). ***, p<0.0005, in a Student's t-test. Representative images are shown in (**ii**).

Zalzman et. al., [25] reported that Zscan4 is key to maintaining the genomic integrity of mouse ESCs. We predicted that if this were the case, then Zscan4 expression may be sensitive to the effects of DNA damage-inducing agents, so we examined whether Zeocin, which induces double strand breaks, or Cisplatin, an alkylating-like agent that induces DNA cross-links leading to double strand breaks via stalled replication forks, affect expression of Zscan4. As shown in Fig. 5A, a 12 h treatment with Zeocin followed by wash-out and incubation for a further 8 and 24 h led to a dose-dependent increase in Zscan4 expression (Fig. 5A (i) and (ii)). In the case of Cisplatin treatment (12 h), a notable enhancement of Zscan4 expression was detected 24 h following wash-out (Figure 5B (i) and (ii)). Induction of γH2AX and the proportion of cells in G2/M phase of the cell cycle were measured to assess the cellular response to these agents (Fig. 5 A and B, panels (i) and (iii)), which showed enhanced γH2AX phosphorylation and accumulation of cells in G2/M phase. Not only were levels of Zscan4 protein enhanced following exposure to Zeocin, but treatment with Zeocin also led to an increase in the proportion of ESCs expressing detectable Zscan4 protein (Fig. 5C(i)), a response partly attenuated by the PI3K inhibitor LY294002 (Fig. 5C(ii)). While the absolute frequency of Zscan4 positive cells varied, due to variation in levels in control populations, we consistently observed a 4–10-fold increase in Zscan4 positive cells following exposure to Zeocin. Phosphorylation of the histone H2AX (to generate γH2AX) is an indicator of DNA damage-induced DNA double strand break and basal levels are known to be elevated in mouse ESCs [51]. However, γH2AX levels are further elevated in ESCs in response DNA damage (see Fig. 5A(i) and B(i)), so we examined the relationship between Zscan4 expression and levels of γH2AX. We found levels of γH2AX were higher in Zscan4 positive cells both under basal (control) conditions and also following treatment with Zeocin (Fig. 5D), consistent with DNA damage corresponding to enhanced Zscan4 expression.

### Zscan4 expression is elevated in late S-phase/early G2 of the cell cycle

**Figure 6:**
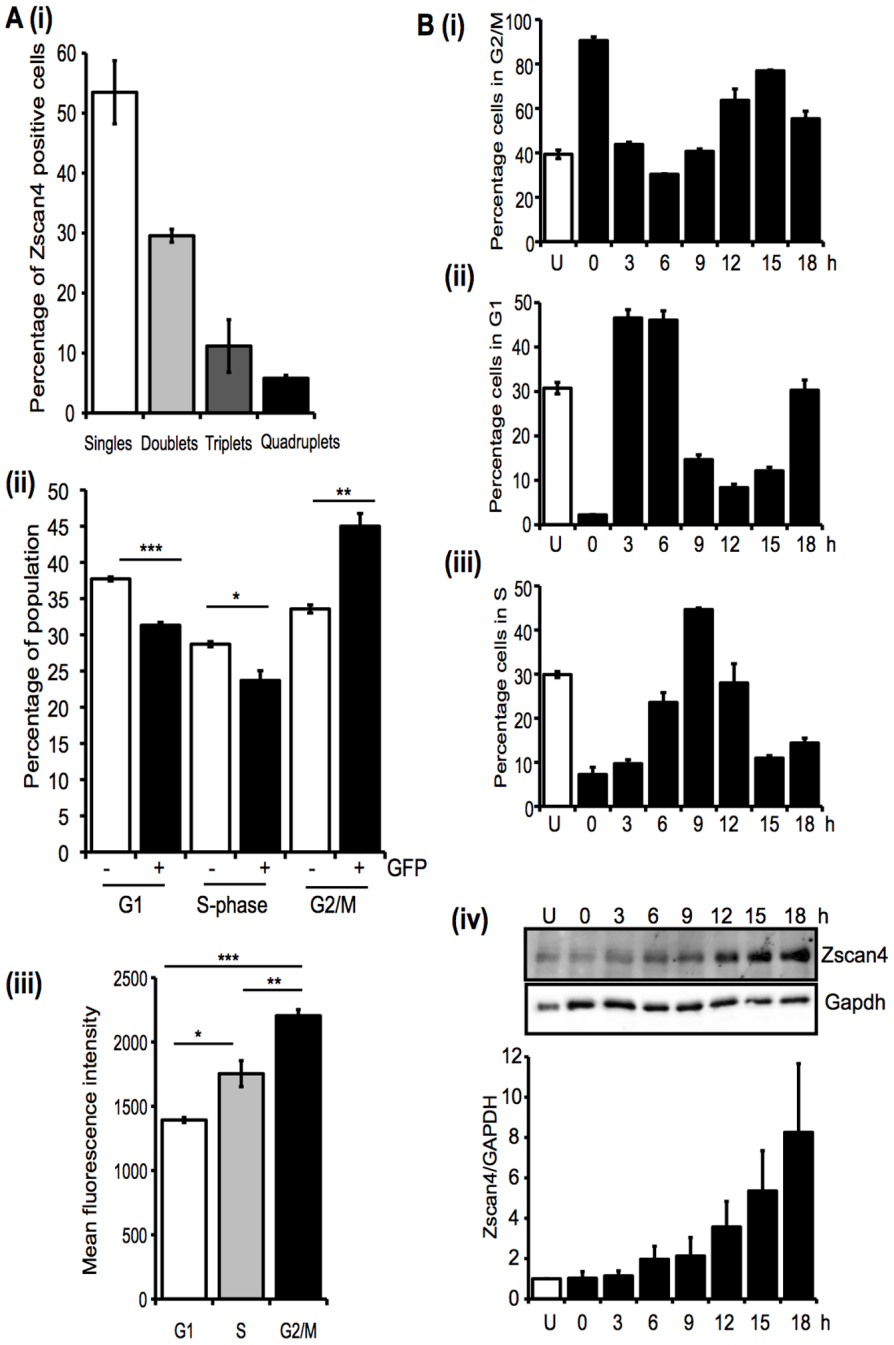
Zscan4 expression is enhanced in late S/early G2 phase of the cell cycle. **A.** (**i**) The proportion of Zscan4 positive cells appearing as individual single cells or in proximity with one, two or three other Zscan4 positive cells were determined. Mean and SEM are shown. Average frequency of Zscan4 positive cells was 0.4%. (**ii**) ZE3-MC1 ESCs were grown in the presence of LIF for 48 h, trypsinised and Nuclear-ID Red (Enzo) used to stain cellular DNA. Cells were analysed by flow cytometry and G1, S and G2/M gates were assigned to the population based on DNA content and using a Nocodazole treated sample as a reference. The cell cycle distribution of GFP negative and positive populations are presented, the mean and SEM are shown (n = 3). *, p<0.05, **, p<0.005, ***, p<0.0005, in a Student's t-test. (**iii**) The mean fluorescence intensity of the GFP positive (Zscan4c expressing) cells distributed in the different cell cycle phases. In each case the mean and SEM are shown. *, p<0.05, **, p<0.005, ***, p<0.0005 following an ANOVA and Tukey's post-hoc test. **B.** E14 ESCs were cultured in LIF and treated with 100 ng/ml Nocodazole for 12 h to induce mitotic arrest. Following release from the block, cell cycle analyses ((**i**), (**ii**) and (**iii**)) and immunoblotting, to detect Zscan4 protein expression (**iv**) were performed at the times indicated (n = 3). Mean and SEM are shown on the graphs. Zscan4 protein expression was normalised to GAPDH ((**iv**), lower panel).

During our analyses of Zscan4 protein expression by immunostaining we noted that positive cells often appeared in close proximity to each other (see Figure 2). Enumerating the proportion of Zscan4 positive cells appearing as single cells, doublets, triplets or quadruplicates (shown in Figure 6A(i)), revealed that approximately 45% of Zscan4 positive cells occur in close proximity to at least one other Zscan4 positive cell. This prompted us to investigate whether Zscan4 proteins were expressed preferentially by cells just prior to or during mitosis. Using the ZE3-MC1 reporter line we investigated the cell cycle distribution profiles of Zscan4 positive and negative cells. As shown in Figure 6A(ii), of all Zscan4 positive cells, approximately 45% were in G2/M phase of the cell cycle, very consistent with our proximity data. Furthermore, the mean fluorescence intensity of Emerald-GFP was highest in cells in G2/M (Figure 6A(iii)), consistent with Zscan4 expression being elevated in cells during these cell cycle phases. To examine this correlation further, we induced mitotic arrest of ESCs using Nocodazole and after release followed both progression through the cell cycle and expression of Zscan4 protein. As shown in Figure 6B(i–iii) approximately 90% of cells were arrested in G2/M by Nocodazole treatment (100 ng/ml). 3 h after release many cells had re-entered G1 (Fig. 6B(ii)), after 6–9 h cells had started to transit through S-phase (Fig. 6B(iii)) and by 12 h were entering G2/M, all consistent with the reported pattern of the ESC cell cycle [52]. Interestingly, Nocodazole treatment alone did not lead to an increase in Zscan4 expression and instead Zscan4 protein levels remained at a constant level until 12 h, when they rose by approximately 4-fold, with the rise continuing to 18 h (Figure 6B(iv)). These data indicate that Zscan4 expression increases as cells transit from late S-phase and into G2 and implicate a selective role for Zscan4 during the G2 checkpoint.

### Zscan4 can modulate transcription and interacts with co-repressor complexes in undifferentiated ESCs

**Figure 7:**
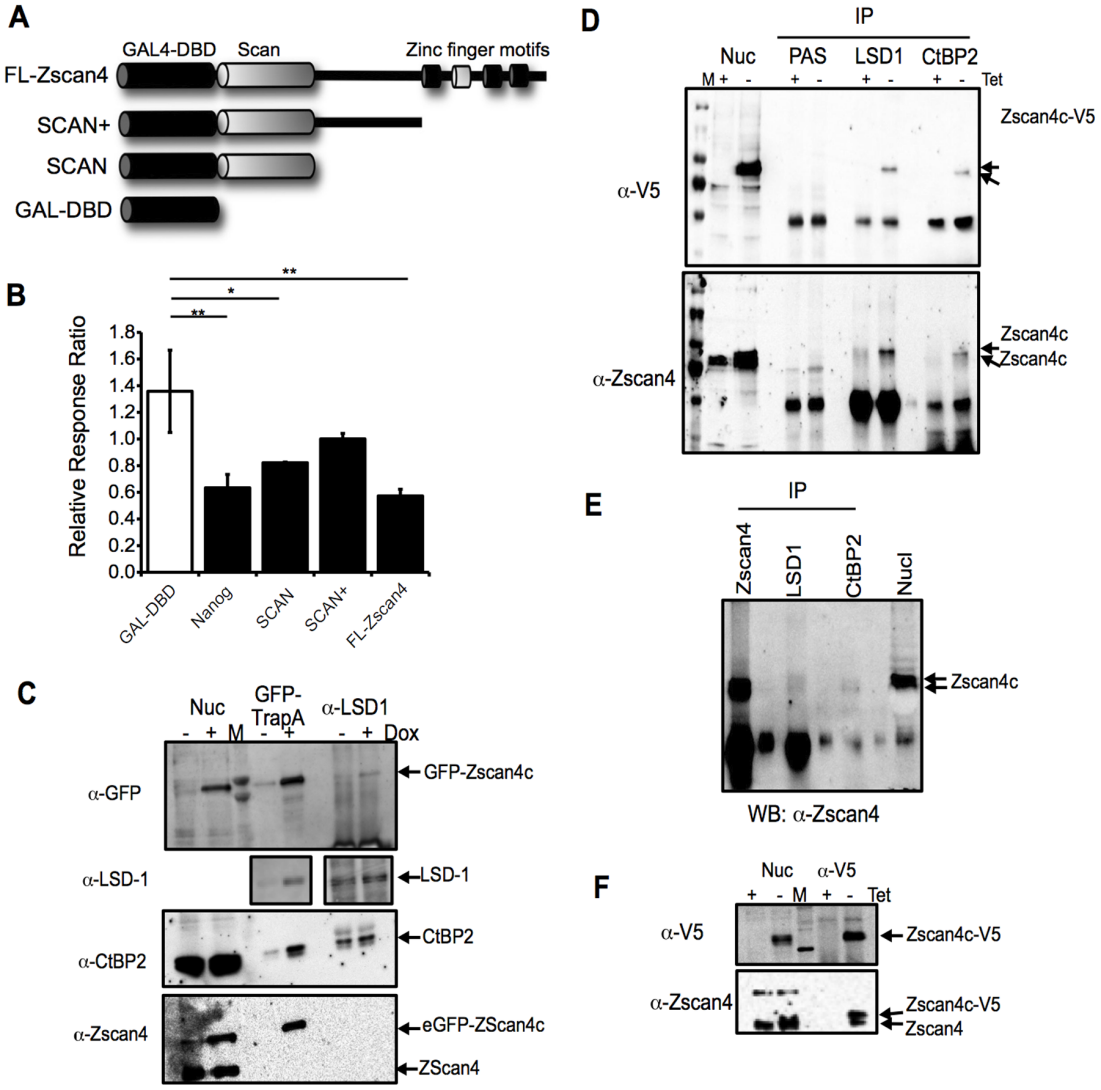
Zscan4 acts as a transcriptional regulator and interacts with LSD-1 and CtBP2. **A.** Schematic showing the structure of GAL4-DNA binding domain fusions with Zscan4c. **B.** Plasmids expressing the GAL-4-DNA binding domain fusions indicated were co-transfected into HEK293 cells along with pGL4-GAL4-UAS plasmid and the pRenilla plasmid (to assess transfection efficiency). 48 h after transfection luciferase activity was determined using Dual-Glo system, according to the manufacturer's recommendations (Promega). Mean and SEM of 3 independent experiments are shown. *, p<0.05, **, p<0.005 following an ANOVA and Tukey's post-hoc test. **C.** ESCs engineered to express an N-Terminal eGFP-Zscan4c fusion protein under the control of the pTRE tight Dox-inducible expression system were cultured in the absence (−) or presence (+) of Dox for 72 h. Nuclear extracts were prepared (Nuc) and immunoprecipitates prepared from 80 µg of protein per sample using either GFP-Trap-A beads or anti-LSD-1 antibodies. Precipitates were divided into two aliquots and separated through 10% polyacrylamide on duplicate gels, prior to immunoblotting. Immunoblotting was performed with anti-GFP, anti-CtBP2 or anti-Zscan4 antibodies, sequentially with one immunoblot. The duplicate immunoblot was probed with anti-LSD-1 antibodies. Positions of precipitated proteins are indicated. **D.** ESCs engineered to express a C-Terminally V5 epitope-tagged version of Zscan4c under the control of the Tet-off expression system were cultured in the presence (+) or absence (−) of Tet for 72 h. Nuclear extracts were made (Nuc) and immunoprecipitates prepared from 80 µg of protein per sample using either anti-LSD-1 or anti-CtBP2 antibodies or protein-A sepharose (PAS) alone as a control. Precipitates were separated through 7.5% polyacrylamide gels prior to immunoblotting. Immunoblotting was first performed with anti-Zscan4 antibodies. The blots were then stripped and reprobed with anti-V5 epitope antibodies. Positions of precipitated proteins are indicated. **E.** Nuclear extracts (Nuc) were prepared from E14 ESCs and precipitates from 200 µg of protein per sample generated using anti-Zscan4, anti-LSD-1 or anti-CtBP2 antibodies. Precipitates were separated through a 6.5% polyacrylamide gel prior to immunoblotting and the lanes between the samples were left blank to avoid the potential of any bleed-through. Immunoblotting was performed with anti-Zscan4 antibodies. Positions of precipitated proteins are indicated. **F.** ESCs engineered to express a C-Terminally V5 epitope-tagged version of Zscan4c under the control of the Tet-off expression system were cultured in the presence (+) or absence (−) of Tet for 72 h. Nuclear extracts were prepared (Nuc) and immunoprecipitates prepared from 80 µg of protein per sample using anti-V5 epitope antibodies. Precipitates were separated through a 7.5% polyacrylamide gel prior to immunoblotting. Immunoblotting was performed with anti-Zscan4 antibodies. Positions of precipitated proteins are indicated.

To understand the mechanism of action of Zscan4 in regulation of ESC fate, particularly how this may relate to a specific role during G2, we investigated the ability of Zscan4 to act as a regulator of transcription. Other Scan-domain and zinc finger containing proteins have been shown to act as transcriptional regulators [53] and while over-expression of Zscan4 has been shown to lead to changes in expression of ∼1000 genes [54], there is no evidence that it acts directly. To examine the ability of Zscan4 to act as a regulator of transcription, we used the GAL4-UAS-luciferase reporter system and generated a series of GAL4-DNA binding domain-Zscan4c fusions, depicted in Fig. 7A. As a control for this assay, we also generated a GAL4-DBD-Nanog fusion. Transient transfection into heterologous HEK293 cells was performed with combinations of the GAL4-UAS-luciferase reporter (containing a synthetic GAL4-UAS promoter, see supplemental Figure S3), GAL4-DBD fusions and Renilla plasmid to control for transfection efficiency. As shown in Fig. 7B, compared to the GAL-DBD alone, fusion of the GAL-DBD with Nanog, the Zscan4c Scan domain alone or full-length Zscan4c, lead to significant decreases in luciferase activity, consistent with transcriptional repression. These data are consistent with Zscan4c playing a direct role in regulating transcription and led us to undertake studies to define Zscan4-interacting proteins in the nucleus of ESCs, which could assist in further definition of the mode of action of Zscan4.

To facilitate purification of Zscan4c interacting proteins, we constructed an eGFP-Zscan4c fusion protein, expressed in R1 ESCs under the control of the pTRE-tight, Dox-inducible expression system (Supplemental Figure S1). Following induction of eGFP-Zscan4c for 24 h, cytosolic and nuclear extracts were prepared and affinity purification performed with GFP-TrapA beads. Induced and un-induced extracts were subjected to the same procedure and after affinity purification and extensive washing protein complexes were released and separated by SDS-PAGE. After staining with Coomassie Blue, protein bands of approximately 116, 100, 75 and 50 kDa, were consistently enriched in the material affinity purified from the induced samples. These proteins were excised and submitted for sequencing at the University of Bristol Proteomics Facility. Following proteolytic digestions with trypsin, tandem mass spectrometry was used to generate peptide mass fingerprints and the Mascot search engine (Matrix Science) exploited to identify proteins. Following these analyses, the 116 kDa protein was identified as the lysine specific demethylase, LSD1, the 100 kDa and 75 kDa proteins as full-length and truncated versions of eGFP-Zscan4c fusion protein respectively and the 50 kDa protein as the transcriptional co-repressor, C-Terminal Binding Protein-2 (CtBP2).

To confirm these Zscan4 protein-protein interactions we first assessed interaction of Zscan4c with LSD-1 and CtBP2 in the eGFP-Zscan4c fusion expressing ESCs. As shown in Figure 7C, the GFP-TrapA beads precipitate the GFP-Zscan4c fusion protein and co-precipitate both LSD-1 and CtBP2 in samples prepared following Dox-induction of eGFP-Zscan4c expression. Furthermore, following immunoprecipitation of LSD-1, eGFP-Zscan4c can be detected in Dox-induced samples. CtBP2 has been reported to form complexes with LSD-1 and we confirm this in mESCs, as CtBP2 is also present in LSD-1 precipitates. We also examined co-precipitation with a version of Zscan4c containing a C-terminal V5 epitope tag. Expression of Zscan4c-V5 is under the control of the Tet-off expression system and as shown in Figure 7D, probing LSD-1 and CtBP2 immunoprecipitates with either anti-V5 epitope or anti-pan Zscan4 antibodies, demonstrates the presence of Zscan4 in those precipitates prepared following Tet-removal, which induces Zscan4c-V5 expression. Interestingly, the Zscan4-V5 precipitating with LSD-1 migrates more slowly than that co-precipitating with CtBP2, suggesting that Zscan4 may be participating in distinct protein complexes and subjected to post-translational modification. This is also apparent when LSD-1 and CtBP2 precipitates are probed with the pan-Zscan4 antibody (Fig. 7D, lower panel) and in these samples there is also evidence for precipitation of endogenous Zscan4 in the +Tet samples which do not over-express Zscan4c-V5. Next we attempted to confirm these interactions in wild-type ESCs, expressing endogenous levels of Zscan4 proteins, a considerable challenge based on our demonstration that less than 1% of the ESC population are expressing Zscan4. As shown in Figure 7E, Zscan4 can be detected in both LSD-1 and CtBP2 precipitates prepared from WT E14 ESCs. Again, LSD-1-associated Zscan4 exhibited slower migration compared to that associated with CtBP2. It has been suggested that the SCAN domain mediates dimerisation [27], [28], [29]. To examine whether this is the case for Zscan4, we precipitated with anti-V5 epitope antibodies and examined if endogenous Zscan4 protein could be co-precipitated. Figure 7F shows that endogenous Zscan4 is present in anti-V5 precipitates, suggesting that exogenously expressed Zscan4c can form dimers with endogenous Zscan4 proteins. Taken together, using three different cell lines, these results confirm that Zscan4 proteins are able to form complexes with LSD-1 and CtBP2 and potentially exist as dimers within ESCs, providing important new insights into the mode of action of Zscan4.

## Discussion

The Zscan4 family of genes have been implicated in the control of early development [26], ESC pluripotency [24] and genome stability/integrity [25], but their regulation and mode of action remain enigmatic. Here we demonstrate that Zscan4c, 4f and ps2 are the most abundant and consistently expressed members of this family, but despite this, expression of Zscan4 protein is restricted to between only 0.1 and 0.7% of ESCs. Significantly, Zscan4 expression was found to be highest in cells transiting late S-phase/early G2 of the cell cycle and we show that DNA damage-inducing agents lead to enhancement of Zscan4 expression, corresponding with accumulation of cells in G2/M. Together these data suggest that Zscan4 plays a selective role in ESCs during G2. Our demonstration that Zscan4 can directly regulate transcription and exists in complexes with LSD-1 and CtBP2 sheds new and important light on its mode of action. These findings lead us to suggest that the Zscan4 family of genes represents an intriguing paradigm for factors that regulate the ESC state.

The very restricted pattern of expression reported for the Zscan4 family is highly distinctive [25], [26], although one caveat to these previous reports has been that they only measured RNA or reporter levels. Our data indicate that the proportion of ESCs within a population expressing Zscan4 protein is between 0.1 and 0.7%, 10-fold lower than the 5% previously reported [25]. Interestingly, we found this level of expression to be consistent across a number of mouse ESC lines and also observed with an iPSC line. We do not think the difference in the proportion of Zscan4 positive cells detected in our study versus previous studies is due to post-transcriptional regulation, since we saw a robust correlation between reporter expression in the ZE3-MC1 line and detection of Zscan4 protein. In addition, this is the same reporter line used by the Ko group [25] but we record fewer Zscan4 positive cells than previously reported. In view of this, it may be that Zscan4 expression is influenced by variation in culture conditions, although when we evaluated this no clear factors emerged. Alternatively, we may have set more stringent thresholds for detection of Zscan4 protein expression, which would have decreased the number of positive cells scored. Nevertheless, it is clear from previous work and the new data that we report here that Zscan4 is expressed by only a small proportion of ESCs in culture. Such a restricted pattern of expression is quite unprecedented in the ESC field and leads to the question of how Zscan4 expression is regulated. Of relevance here is a recent study that has reported the identification of a rare and transient population of cells within ESC cultures that share properties and expression profiles with two-cell stage embryos [55]. This is interesting from the point of view of the Zscan4 family, since Zscan4d was first identified as a gene whose expression was elevated during zygotic genome activation and highly expressed in 2-cell stage embryos [26]. Macfarlan et al., report that many of the ‘2C’ transcripts detected in this transient sub-population of ESCs are initiated from the LTRs of endogenous retroviruses, including those of Zscan4 [55]. Thus, it is possible that activation of these endogenous retroviral elements play a role in establishing the highly restricted pattern of expression of Zscan4 proteins within ESC populations. Furthermore, it is tempting to speculate that Zscan4 is a marker of this rare and transient ‘2C’ stem cell population and given that RNAi knock-down of Zscan4 leads to arrest of 2-cell embryo development [26], it could play an important functional role in this rare cell population. However, alternative explanations for the restricted pattern of expression of Zscan4 also need to be considered and explored, including auto-regulatory or oscillatory-based mechanisms. Further work will be required to distinguish between these possibilities.

Based on the evidence published to date [24], [25], [26], [33], [35], [55], it seems likely that Zscan4 plays multiple roles in pluripotent cells and we were particularly interested to discover that Zscan4 expression varied at different stages of the cell cycle. Initially our observation that 45% of Zscan4 positive cells were found in close proximity to another Zscan4 positive cell led us to consider that Zscan4 expression may be enriched in cells undergoing mitosis. However, when ESCs were treated with Nocodazole, which leads to arrest in pro-metaphase, Zscan4 expression was not elevated. Instead, upon release and progression through the cell cycle, increases in Zscan4 protein levels corresponded with the stage at which cells transited late S-phase and entered early G2. Further support for a role of Zscan4 in G2 phase of the cell cycle comes from our studies examining regulation of Zscan4 expression in response to DNA damaging agents. Both Zeocin and Cisplatin induce double-stranded DNA breaks, as measured by an increase in γH2AX, which lead to cell cycle arrest in G2/M [56]. We detected the highest levels of Zscan4 induction by Zeocin at the point when the number of cells in G2/M had increased from 40 to 65%. In the case of Cisplatin, the most significant elevation in Zscan4 expression was observed with the highest dose of Cisplatin we used following a 24 h recovery phase. At this same time point, the proportion of cells in G2/M had increased from 40 to 57%. While we had initially assessed cell cycle profiles of these cells to gauge the ESC response to DNA damage-inducing agents, these data provide additional evidence to support the fact that Zscan4 expression is preferentially increased during G2-phase of the cell cycle. Furthermore, it is worth noting that temporal changes in Zscan4 expression do not follow the profile of γH2AX detection, indicating Zscan4 up-regulation is not an early response to DNA damage. Given the report that Zscan4 plays a critical role in telomere elongation and genome integrity in mouse ESCs [25] our demonstration that agents that damage DNA lead to an enhancement in Zscan4 expression is consistent with a role for Zscan4 in genome surveillance and maintenance.

The fact that members of the Zscan4 family contain zinc finger domains has led to the suggestion that they act as transcription factors [24], as do other members of the SCAN-domain containing family [53]. Over-expression of Zscan4 in mouse ESCs has been reported to result in the change in expression of over 1000 genes [54], while inclusion of Zscan4 as a reprogramming factor for mouse embryo fibroblasts results in the transient induction of pre-implantation specific genes [33]. Despite these reports, evidence to support the ability of Zscan4 to directly regulate transcription has been lacking. Using a heterologous reporter system, we demonstrate that Zscan4 can act as a transcriptional regulator. Both full-length Zscan4 and the SCAN domain alone were able to repress GAL4 binding domain-mediated reporter expression (Figure 7B). This could be due to the ability of the SCAN domain to promote dimerisation, as demonstrated for other SCAN-domain-containing proteins [28], [29]. Importantly, we provide the first evidence that Zscan4 may act as a dimer, since we show that a V5-epitope-tagged version of Zscan4 co-precipiates endogenous Zscan4 proteins (Figure 7F). To gain further insight into the mode of action of Zscan4, we investigated whether Zscan4 interacted with other proteins within ESC nuclei. Our analyses revealed two major interacting proteins -LSD-1 and CtBP2. LSD1 has been reported to specifically demethylate mono and di-methyl histone H3 at K4 and K9 and thus acts as a co-repressor of transcription. CtBP2 belongs to a class of transcriptional co-repressors that bind to transcription factors via a PXDLS motif and has been shown to interact with a range of Zinc finger containing transcriptional regulators [57], [58]. Zscan4c exhibits a PXDLS motif at position 263–268, as do Zscan4 family members, suggesting that each of them can bind CtBP2. Importantly, both LSD-1 and CtBP2 have been reported to be components of the CoREST-CtBP repressor complex [58], [59]. Thus, the identification of these two proteins as Zscan4c interacting partners in ESCs is consistent with the fact that Zscan4c is a putative regulator of transcription, possibly involved in repression of transcription. It has been suggested that the fact Zscan4 over-expression leads to minor changes in the transcriptome during early reprogramming events, but major differences in iPSC outcome, indicates it acts differently to many other factors and may be involved in epigenetic regulation or chromatin remodeling [33]. Our finding that Zscan4 interacts with LSD-1 is consistent with this latter possibility. In future studies it will be interesting to determine the genes that are directly targeted by Zscan4 in ESC and iPSCs and compare these to known targets of LSD-1 and CtBP2.

Our expression data indicate that three Zscan4 genes are most abundantly expressed in ESCs, namely Zscan4c, 4f and ps2. Zscan4-ps2 has previously been characterized as a pseudogene, primarily based on the fact that in a previous study no ps2 transcripts were detected in mouse ESCs [26], although in contrast our data indicate Zscan4-ps2 transcripts can be detected in mouse ESCs. Given the close relationship between Zscan4c, 4f and ps2 (Fig. 1B), it is intriguing that these should consistently be the most highly expressed in different ESCs lines, but argues for a predominant role of their gene products. Also worth noting is the fact that the p110α isoform of PI3Ks plays the predominant role in regulating Zscan4 expression, supported by both loss and gain of function studies (Figure 3). We have previously implicated p110α PI3K in regulation of ESC proliferation [48], [60], which complements with the findings we report here relating to Zscan4 expression and the cell cycle. PI3K signaling is one of the pathways that regulate activity of Gsk-3. Surprisingly, given its role in the ground state of ESC pluripotency, short-term inhibition of Gsk-3-dependent signaling did not lead to increased Zscan4 protein expression, whereas a sustained lack of Gsk-3 activity did. However, no significant increase in the proportion of Zscan4 positive cells was detected in Gsk-3 double knock-out ESCs (data not shown).

Based on previously published studies and the results we present here, we would like to suggest that the Zscan4 family represents an intriguing paradigm for regulators of the for ESC state. Multiple, closely related Zscan4 genes are co-expressed by ESCs, but despite this Zscan4 protein is detected in less than 1% of ESCs. Zscan4 expression is higher in cells in late S/early G2-phase of the cell cycle, consistent with it playing a role during the G2 checkpoint and genome stability. Related to this we have demonstrated that Zscan4 acts as a regulator of transcription and interacts with LSD-1 and CtBP2, suggesting it may have a role in transcriptional repression. While many questions remain to be answered about this enigmatic family of zinc finger proteins, our study provides new and important insight into the regulation and action of Zscan4 proteins.

## Supporting Information

Figure S1 **Establishment and characterisation of eGFP-Zscan4c-Tet-on inducible ESC lines.** **A.** Schematic of the GFP-Zscan4c fusion protein generated. **B.** Following induction with 1 mg/ml doxycycline for the times indicated (in hours, h), nuclear lysates were prepared from eGFP-Zscan4c ESCs and immunoblotted with an anti-GFP antibody. Blots were stripped and re-probed with an anti-TBP antibody to assess the equality of protein loading. **C.** Quantitative RT-PCR was used to confirm induction of Zscan4c expression normalised to β-actin levels. **D.** Fluorescent images showing expression of eGFP-Zscan4c (green) after addition of doxycycline for the times indicated.
(TIF)

Figure S2 **Establishment and characterisation of Zscan4c-V5-His Tet-off inducible ESC lines.** **A.** Immunoblots showing induction of Zscan4c-V5-His protein (clone 43) upon withdrawal of tetracycline (tet) detected with anti-V5 antibody. (**i**) Expression 24 h after induction in the presence of different concentrations of LIF (indicated in U/ml). (**ii**) Expression of Zscan4c-V5-His was maintained over a time-course of 120 h. Immunoblots were stripped and re-probed with Oct-4 antibody. **B.** (**i**) Localisation of Zscan4c-V5-His protein was assessed by immunostaining of Tet-off inducible cell lines grown in the absence of tetracycline. Anti-V5 antibody was used to detect Zscan4c-V5 protein (red). Cell nuclei were counter-stained with DAPI (blue). (**ii**) Zscan4c-V5-His-Tet-off inducible mESC (clone 45) were grown in the presence and absence of Tet. Cytosolic and nuclear proteins were separated and immunoblotting performed with anti-V5, anti-TBP (predominantly nuclear) and anti-GAPDH (predominantly cytosolic) antibodies.
(TIF)

Figure S3 The sequence (5′-3′) of the synthetic GAL4-UAS promoter used to drive luciferase expression in this study. The strands were mixed, heated to 95°C and cooled before being ligated into pGL4.26 (Promega).
(TIF)

Figure S4 **Zscan4 antibody specificity.** ESCs engineered to express a C-terminally V5 epitope-tagged version of Zscan4c under the control of the Tet-off expression system were cultured in the presence (+) or absence (−) of Tet for 48 h when nuclear (Nuc) and cytoplasmic (Cyto) protein extracts were prepared. **A.** Cytosolic and nuclear extracts were separated by SDS-PAGE and duplicate blots prepared. A 1∶2000 dilution of anti-Zscan4 anti-peptide antibody was incubated with or without 1 µg/ml blocking peptide for 1 h prior to being used for immunoblotting. **B.** Immunoprecipitates were prepared from (**i**) 1 mg of cytosolic or (**ii**) 80 µg of nuclear protein extract per sample using either protein-A sepharose (PAS) bead alone or together with 2 µg anti-Zscan4 antibody. Precipitates were separated through 7.5% polyacrylamide gels prior to immunoblotting. Immunoblotting was performed with anti-V5 epitope (upper panels) or anti-Zscan4 (lower panels) antibodies. Positions of precipitated proteins are indicated by the arrows.
(TIF)

Figure S5 **Assigning Zscan4 gene transcripts.** The transcript sequence from each Zscan4 gene was obtained from the NCBI database. These were aligned and distinguished based on the identification of SNPs – highlighted in black in the schematic. Sequenced Zscan4 cDNAs derived from E14tg2a or CCE cell lines (numbers assigned based on position in 96-well tray, e.g. E11, C07 as shown) were edited and added to this alignment. Sequences were assigned to a Zscan4 gene based on the distinguishing SNPs each contained. As an example, the 5 SNPs used to identify Zscan4a transcripts are shown. Sequence data were edited and aligned using Sequencher (GeneCodes).
(TIF)

Figure S6 **Regulation of Zscan4 by the p110a catalytic subunit of PI3K.** E14tg2a cells were treated with either **A.** 5 µM LY294002 or 600 nM Compound 15e or **B.** 5 µM LY294002, 10 µM TGX-121, 5 µM IC87114 or 1 nM Rapamycin. RNA was extracted 48 h after inhibitor treatment, quantitative RT-PCR was performed and Zscan4 expression normalised relative to β-actin levels. Graphs show standard deviation and are representative of three experimental repeats.
(TIF)

Table S1 Primer sequences used for quantitative RT-PCR.
(DOCX)

Table S2 Primer sequences used for construction of Gal4-Zscan4 fusion transcriptional reporters.
(DOCX)
