# Supplementary figures and images for: Zscan4 Is Regulated by PI3-Kinase and DNA-Damaging Agents and Directly Interacts with the Transcriptional Repressors LSD1 and CtBP2 in Mouse Embryonic Stem Cells
Source: PLoS One. 2014 Mar 3;9(3):e89821. doi: 10.1371/journal.pone.0089821 (PMC3940611; doi:10.1371/journal.pone.0089821)

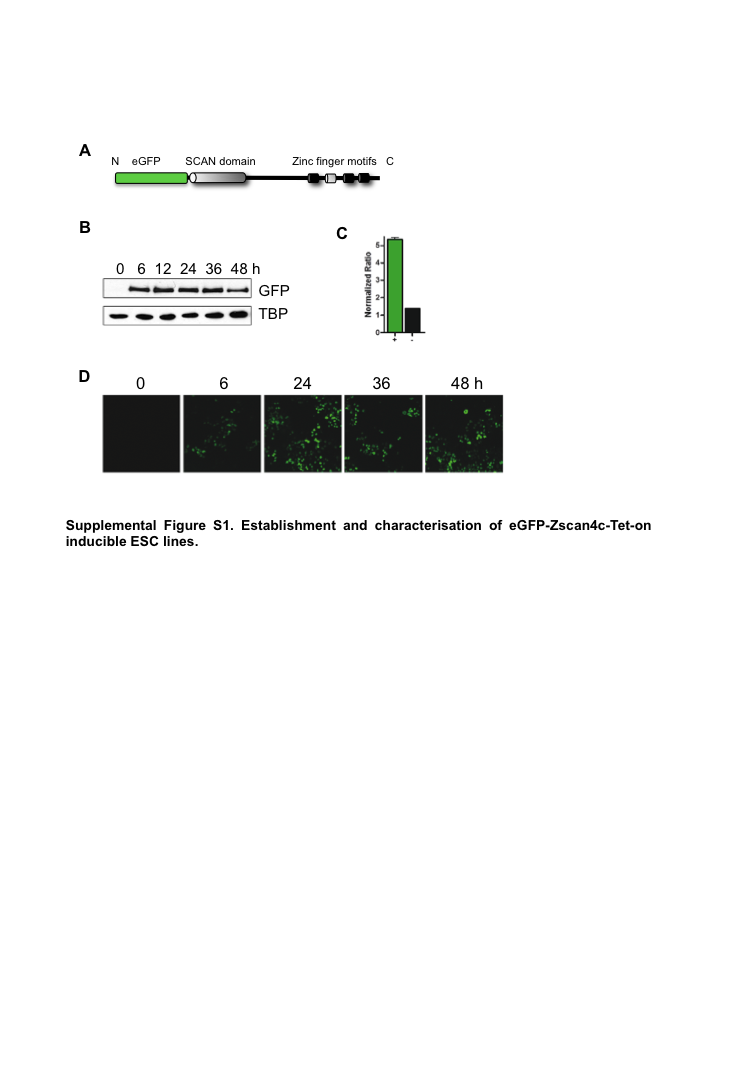

Supplement: Figure S1 — Establishment and characterisation of eGFP-Zscan4c-Tet-on inducible ESC lines. A. Schematic of the GFP-Zscan4c fusion protein generated. B. Following induction with 1 mg/ml doxycycline for the times indicated (in hours, h), nuclear lysates were prepared from eGFP-Zscan4c ESCs and immunoblotted with an anti-GFP antibody. Blots were stripped and re-probed with an anti-TBP antibody to assess the equality of protein loading. C. Quantitative RT-PCR was used to confirm induction of Zscan4c expression normalised to β-actin levels. D. Fluorescent images showing expression of eGFP-Zscan4c (green) after addition of doxycycline for the times indicated. (TIF) [file pone.0089821.s001.tif]

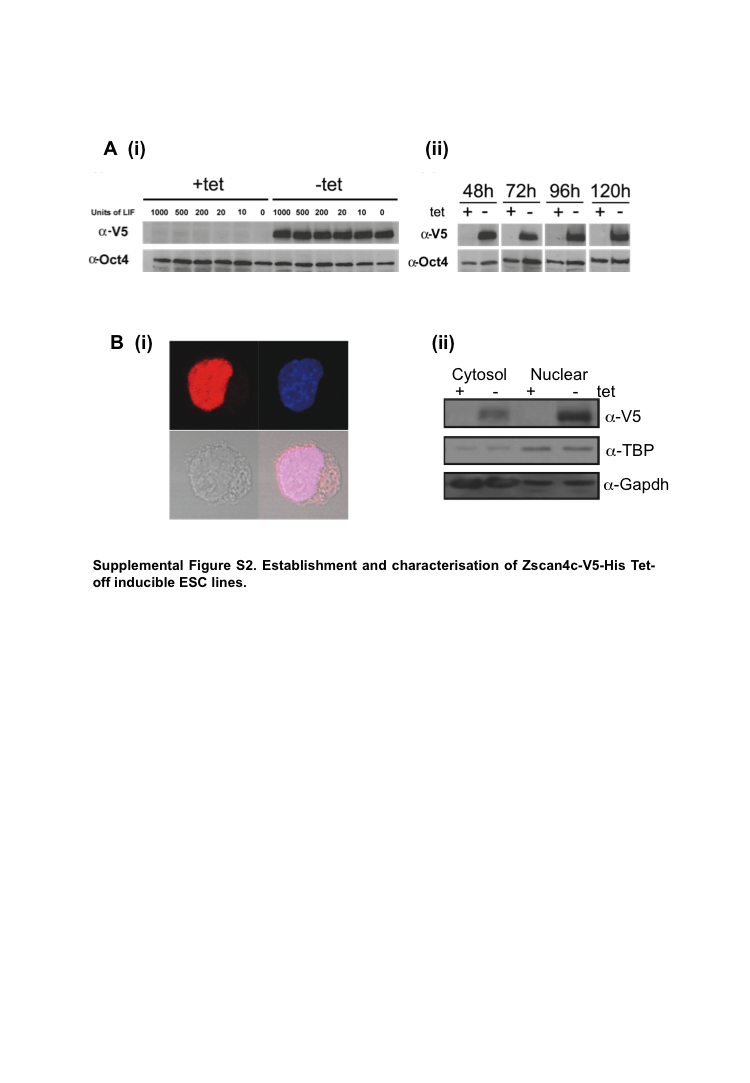

Supplement: Figure S2 — Establishment and characterisation of Zscan4c-V5-His Tet-off inducible ESC lines. A. Immunoblots showing induction of Zscan4c-V5-His protein (clone 43) upon withdrawal of tetracycline (tet) detected with anti-V5 antibody. (i) Expression 24 h after induction in the presence of different concentrations of LIF (indicated in U/ml). (ii) Expression of Zscan4c-V5-His was maintained over a time-course of 120 h. Immunoblots were stripped and re-probed with Oct-4 antibody. B. (i) Localisation of Zscan4c-V5-His protein was assessed by immunostaining of Tet-off inducible cell lines grown in the absence of tetracycline. Anti-V5 antibody was used to detect Zscan4c-V5 protein (red). Cell nuclei were counter-stained with DAPI (blue). (ii) Zscan4c-V5-His-Tet-off inducible mESC (clone 45) were grown in the presence and absence of Tet. Cytosolic and nuclear proteins were separated and immunoblotting performed with anti-V5, anti-TBP (predominantly nuclear) and anti-GAPDH (predominantly cytosolic) antibodies. (TIF) [file pone.0089821.s002.tif]

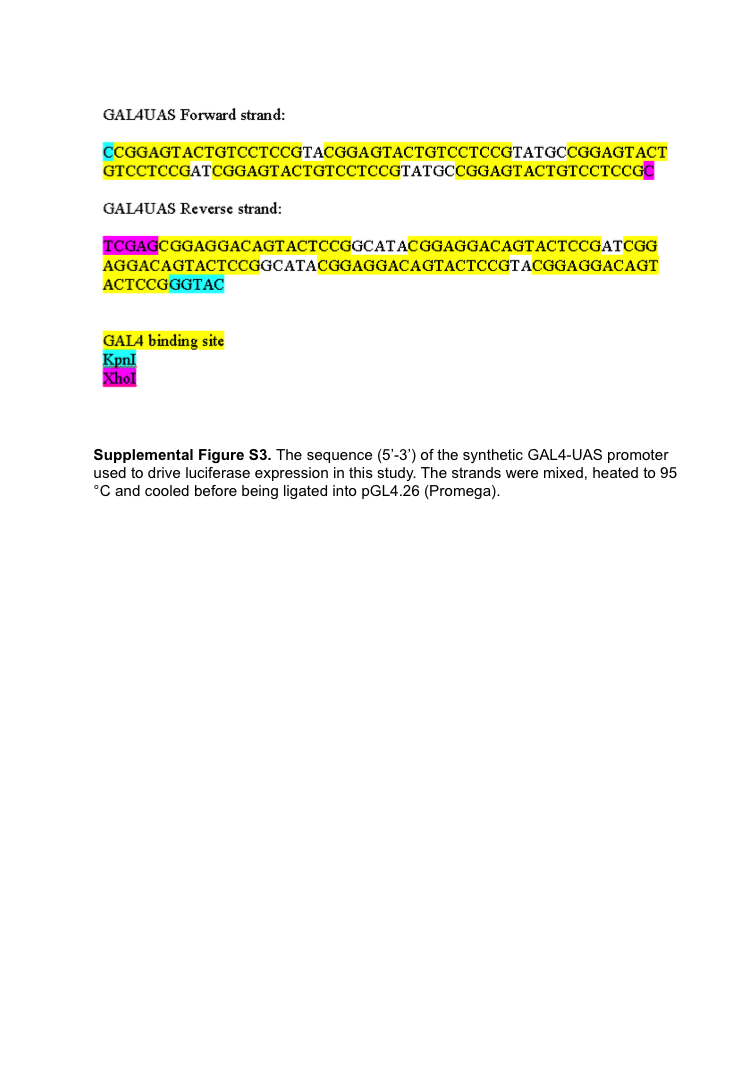

Supplement: Figure S3 — The sequence (5′-3′) of the synthetic GAL4-UAS promoter used to drive luciferase expression in this study. The strands were mixed, heated to 95°C and cooled before being ligated into pGL4.26 (Promega). (TIF) [file pone.0089821.s003.tif]

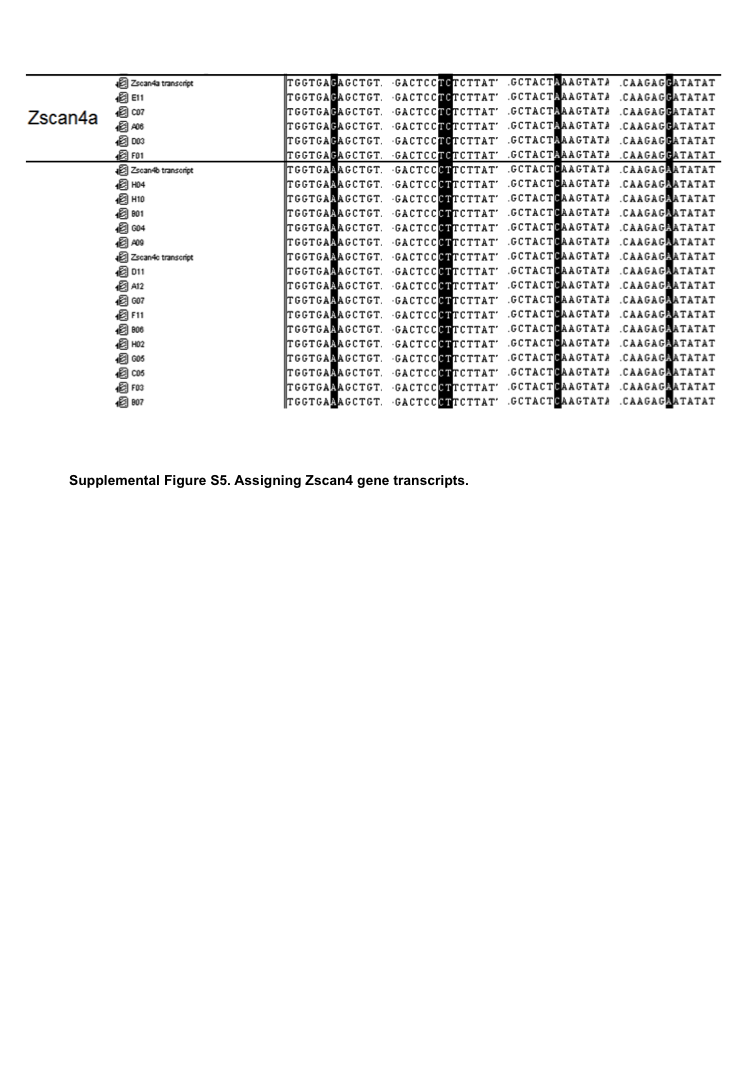

Supplement: Figure S5 — Assigning Zscan4 gene transcripts. The transcript sequence from each Zscan4 gene was obtained from the NCBI database. These were aligned and distinguished based on the identification of SNPs – highlighted in black in the schematic. Sequenced Zscan4 cDNAs derived from E14tg2a or CCE cell lines (numbers assigned based on position in 96-well tray, e.g. E11, C07 as shown) were edited and added to this alignment. Sequences were assigned to a Zscan4 gene based on the distinguishing SNPs each contained. As an example, the 5 SNPs used to identify Zscan4a transcripts are shown. Sequence data were edited and aligned using Sequencher (GeneCodes). (TIF) [file pone.0089821.s005.tif]

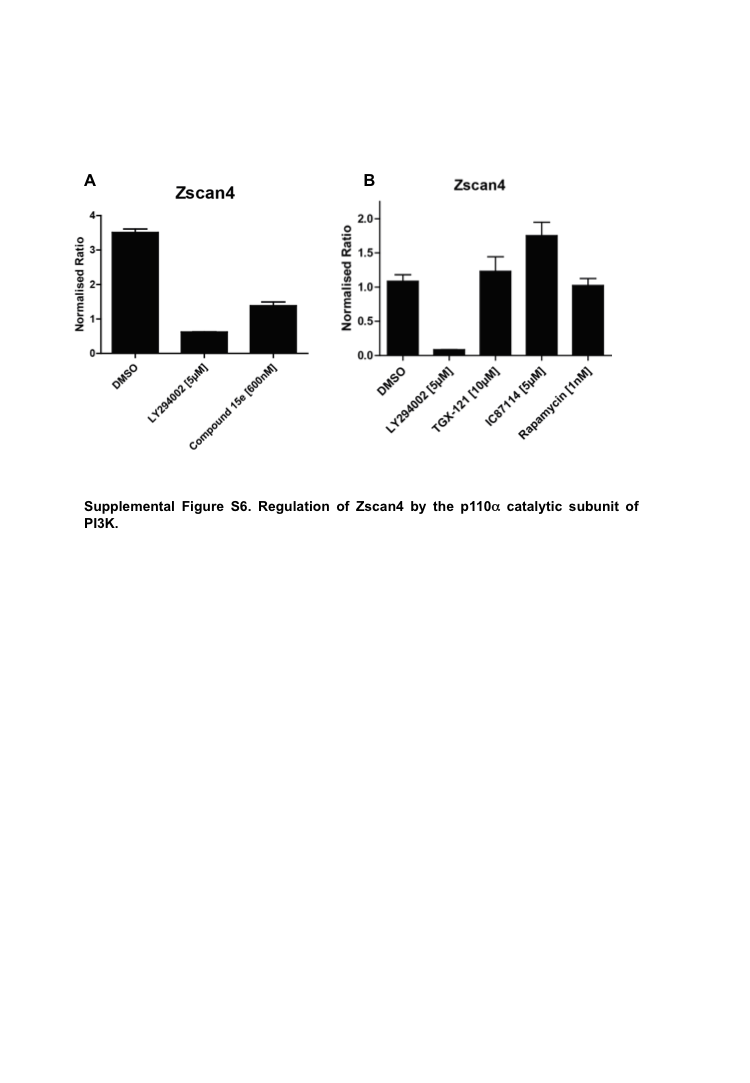

Supplement: Figure S6 — Regulation of Zscan4 by the p110a catalytic subunit of PI3K. E14tg2a cells were treated with either A. 5 µM LY294002 or 600 nM Compound 15e or B. 5 µM LY294002, 10 µM TGX-121, 5 µM IC87114 or 1 nM Rapamycin. RNA was extracted 48 h after inhibitor treatment, quantitative RT-PCR was performed and Zscan4 expression normalised relative to β-actin levels. Graphs show standard deviation and are representative of three experimental repeats. (TIF) [file pone.0089821.s006.tif]
