# Supplementary material for: Zscan4 Is Regulated by PI3-Kinase and DNA-Damaging Agents and Directly Interacts with the Transcriptional Repressors LSD1 and CtBP2 in Mouse Embryonic Stem Cells
Source: PLoS One. 2014 Mar 3;9(3):e89821. doi: 10.1371/journal.pone.0089821 (PMC3940611; doi:10.1371/journal.pone.0089821)
Supplement: Table S1 — Primer sequences used for quantitative RT-PCR. (DOCX) [file pone.0089821.s007.docx]

**Supplementary Table S1.** Primer sequences used for quantitative RT-PCR.

| **Gene** |  | **Sequence (5'-3')** | **Annealing Temperature** |
| --- | --- | --- | --- |
| Zscan4 | Forward | TTGAAGCCTCCTGTCATGGTCC | 61°C |
|  | Reverse | TCCATTTCATTTCCACTACAGC |  |
| β-actin | Forward | TAGGCACCAGGGTGTGATGG | 60°C |
|  | Reverse | CATGGCTGGGGTGTTGAAGG |  |
| GAPDH | Forward | ACCACAGTCCATGCATCAC | 58°C |
|  | Reverse | TCCACCACCCTGTTGCTGTA |  |
