# Supplementary material for: Zscan4 Is Regulated by PI3-Kinase and DNA-Damaging Agents and Directly Interacts with the Transcriptional Repressors LSD1 and CtBP2 in Mouse Embryonic Stem Cells
Source: PLoS One. 2014 Mar 3;9(3):e89821. doi: 10.1371/journal.pone.0089821 (PMC3940611; doi:10.1371/journal.pone.0089821)
Supplement: Table S2 — Primer sequences used for construction of Gal4-Zscan4 fusion transcriptional reporters. (DOCX) [file pone.0089821.s008.docx]

**Supplementary Table S2.** Primer sequences used for construction of Gal4-Zscan4 fusion transcriptional reporters.

| **Gene** | **Fusion protein and amino acids from fusion partner** |  | **Sequence (5'-3')** |
| --- | --- | --- | --- |
| Zscan4 | BamHI Gal4 Fus | Forward | ATAAGGATCCGCTTCACAGCAGGCACCAGC |
|  | EcoRI SCAN Fus (A2-F137) | Reverse | AAAAGAATTCGAAAAGAGGGCTTCTTGTCC |
|  | EcoRI SCAN+ Fus (A2-K393) | Reverse | ATTTGAATTCTTTGGCATCTCTACAGAATCTC |
|  | EcoRI SCAN+ZnF Fus (A2-R504) | Reverse | TTTAGAATTCATCTGTGGTAATTCCTCAGG |
| Nanog | BamHI Nanog (N22-N296) | Forward(2) | ATAAGGATCCAACGCCTCATCAATGCCTGC |
|  | EcoRI Nanog | Reverse | AATTTGAATTCGTTCAGGAATAATTCCAAGG |
| Oct4 | BamHI Oct4 (G45-A344) | Forward(2) | ATAAGGATCCGGAATCGGACCAGGCTCAGAGG |
|  | EcoRI Oct4 | Reverse | AATTTGAATTCGAGCAGTGACGGGAACAGAGG |
